# Supplementary material for: Risk factors for disruptive behaviours: protocol for a systematic review and meta-analysis of quasi-experimental evidence
Source: BMJ Open. 2020 Sep 9;10(9):e038258. doi: 10.1136/bmjopen-2020-038258 (PMC7482491; doi:10.1136/bmjopen-2020-038258)
Supplement: Supplementary data [file bmjopen-2020-038258supp001.pdf]

Appendix A: Summary of Terms

Concept 1 – Quasi-experimental terms separated into quasi-experimental study designs, quasi-experimental statistical methods and quasi-experimental features.

Term	Definition
**Quasi-experimental study designs**	
Adoption study	Adoption separates genetically related parents and children and places children in a different rearing environment. Adoption studies compare associations between exposures and outcomes in parents and their children that are genetically related (not adopted) and genetically unrelated (adopted) to them. [1]
In vitro fertilisation study	In vitro fertilization can either use either parental gametes (genetically related) or donor gametes (genetically unrelated) for fertilization. Similar to adoption studies, in vitro fertilisation studies compare associations between exposures and outcomes in parents and their children that are genetically related and genetically unrelated. [1]
Genetically informed methods	Study designs or statistical analyses that use genetic information (e.g. known genetic relationships between twins) or data on genetic variations. [1]
Natural experiment	Natural experiments use natural randomly occurring circumstances (e.g. lottery win, policy or law change) as an exposure, which is assigned "as random". [2] True natural experiments are very unusual therefore they are usually described as "quasi natural experiments" or observational studies with exogenous exposures.
Quasi-experimental study	Quasi-experimental studies assess causality by aiming to replicate the counterfactual framework, also known as the potential outcomes framework. Quasi-experimental studies aim to reproduce the counterfactual framework either by design (e.g. natural experiments or twin studies) or by employing statistical methods (e.g. propensity score matching or difference-in-difference studies). These methods fall into two broad categories according to whether they invoke the assumption of no unmeasured confounding (e.g. g-methods) or exploit the presence of instrumental variables (e.g. Mendelian randomisation).
Sibling study	On average, siblings share 50% of their segregated genetic material. Similar to twin studies, some sibling studies compare outcomes in exposed versus non-exposed siblings. [1]
Twin study	On average, dizygotic twin pairs share 50% of their segregated genes compared to monozygotic twin pairs which share 100% of their genetic material. A twin that is non-exposed to a risk factor represents a natural match to their exposed co-twin. Therefore, some twin studies compare outcomes in exposed versus non-exposed pair members. [1]
**Quasi-experimental statistical methods**	
Difference in difference study / controlled before and after study	Difference-in-Differences designs, also known as controlled before and after studies, are a type of fixed effect study. The difference before and after the exposure in the exposed group is compared to the same period of time in the non-exposed group. The exposure effect is the difference between these differences. If an exposure has a harmful effect, the outcome will occur more rapidly in individuals that receive the exposure than in individuals that do not receive the exposure. [7]

Fixed effects	Applied to longitudinal data with repeated measures, fixed effects methods model within-individual changes over time (i.e. variation in an individual's exposures and outcomes), as opposed to between-individual changes (i.e. variation across individuals), to remove time-invariant confounding, with each individual acting as their own control. Difference-in-difference, controlled before-and-after, experience sample and ecological momentary assessment are all examples of fixed effect analyses [3].
Instrumental variable analysis	Analyses that use variables that are associated with an exposure of interest (assumption 1), do not share any common causes with the outcome (assumption 2) and affect the outcome only through the exposure (assumption 3), also known as instrumental variables. These variables can be any traits that meet the three instrumental variable assumptions, for example genetic variants (e.g. Mendelian randomisation). [5]
Interrupted time series analysis	Interrupted time series methods use observational data collected over equally spaced intervals before and after an intervention, that is exogenous to the time series, e.g. a "natural experiment" in the real word setting. The effect of the intervention is evaluated by examining whether the data pattern (e.g. the level and slope) observed post-intervention is different to that observed pre-intervention. [6]
Regression discontinuity analysis	If treatment allocation is based on whether a patient scores below or above a predetermined cut-off value, as opposed to randomisation, then the intervention will be randomly assigned for patients close to the threshold. [4] See also "Sharp/fuzzy design" below.
**Quasi-experimental features**	
Causal effect	An exposure has a causal effect on the outcome if the outcome differs when the exposure is present compared to when the exposure is absent, all other things being equal. [8]
Counterfactual framework	The comparison of hypothetical scenarios whereby the *same* individual is either exposed or unexposed to a risk factor. Also known as the potential outcomes framework. [8–10]
Doubly robust estimation	Doubly robust estimation combines two models: outcome regression and propensity score modelling. Individually these two methods lead to unbiased estimators of the causal effect only if the respective model is correctly specified; when they are combined, through doubly robust estimation, only one of the two models needs to be correctly specified to obtain an unbiased effect estimator. [11]
Heckit model/Heckman sample selection	Similar to selection/selectivity models, these are used to handle non-ignorable missing data. Heckit/Heckman selection models assume (a) a joint distribution for the missingness and outcome processes and (b) validity in the instrument. If these assumptions are met, these models can correct bias from non-randomly selected samples.
Matching study	Researchers can attempt to create a reasonable counterfactual by accounting for confounders via matching exposed and non-exposed participants on key variables. Propensity score matching approaches can be select appropriate matches (either to cases or non-cases or both) leading to different causal effects. Quasi-experimental designs are often combined with propensity score matching approaches. [2] See "Propensity score"
Potential outcome	The outcome that would occur had the exposure been set to a particular value; see also "Counterfactual framework".

Propensity score	A propensity score is the probability of being exposed conditional on the confounders. It has the advantage of reducing, a potentially large, number of confounders into a scalar that contains all information that is relevant for the exposure assignment in relation to the outcome. The propensity score is used as an additional covariate in outcome regression, or as a stratifying or matching variable. Inverse probability weights derived from propensity scores also remove confounding by recreating a pseudo-sample where there is no confounding. See "Matching study".
Selection/selectivity model	A model that deals with samples that are non-randomly selected, and therefore non-representative of the target population. For example, studies affected by non-ignorable missing data.
Sharp design/Fuzzy design	These are features of regression discontinuity designs.<br><br>A sharp discontinuity regression design exploits exogenous changes to the value of an exposure /intervention to estimate its causal effect on an outcome. These changes are usually triggered by overtaking a particular (sharp) threshold in a continuous endogenous variable. Since the comparison with the threshold may be affected by random error, individuals with values near the threshold can be viewed as being "as good as" randomly allocated to the exposure and analysed as if they were in an RCT.<br><br>In a fuzzy regression discontinuity design, the threshold does not need to be defined as a sharp discontinuity as long as the probability of exposure/intervention assignment differs among those near the threshold.

Concept 2 – Disruptive behaviour terms

Term	Definition
Disruptive behaviour disorders / externalising disorders	A range of repetitive and troublesome behaviours, such as lying, fighting and stealing. It can include one or more of the below behaviours and the term is sometimes used interchangeably with "externalising disorders".
Antisocial personality disorder (DSM) / dissocial personality disorder (ICD)	A diagnosis which involves a life-long pattern of antisocial behaviour as well as irritability and remorselessness. By definition, a diagnosis of ASPD involves exhibiting conduct disorder in childhood.
Conduct disorders	A formal diagnosis whereby an individual displays repetitive and persistent patterns of antisocial, aggressive or defiant behaviour that amounts to significant and persistent violations of age-appropriate social expectations [12] and are diagnosable as defined by the DSM-5.
Conduct problems	An umbrella term to describe a range of repetitive and disruptive behaviours, such as lying, fighting and stealing that do not necessarily meet the threshold for diagnosis of conduct disorder.

Oppositional defiant disorder	A formal diagnosis whereby an individual exhibits defiant and disobedient behaviour towards others as opposed to conduct disorder, whereby behaviours violate the rights of others and/or societal expectations.
Psychopathy	Psychopathy is characterised by high levels of antisocial behaviour, low levels of anxiety and high levels of attention seeking. It is a specifier for antisocial personality disorder in the DSM-5.

References

1 Pingault J-B, O'Reilly PF, Schoeler T, *et al.* Using genetic data to strengthen causal inference in observational research. *Nat Rev Genet* 2018;**19**:566–80. doi:10.1038/s41576-018-0020-3

2 Rutter M. 'Natural Experiments' as a Means of Testing Causal Inferences. In: *Causality: Wiley Series in Probability and Statistics*. 2012. 253–72. doi:10.1002/9781119945710.ch18

3 Gunasekara FI, Richardson K, Carter K, *et al.* Fixed effects analysis of repeated measures data. *Int J Epidemiol* 2014;**43**:264–9. doi:10.1093/ije/dyt221

4 Bor J, Moscoe E, Mutevedzi P, *et al.* Regression Discontinuity Designs in Epidemiology. *Epidemiology* 2014;**25**:729–37. doi:10.1097/EDE.0000000000000138

5 Davies NM, Holmes M V., Davey Smith G. Reading Mendelian randomisation studies: a guide, glossary, and checklist for clinicians. *BMJ* 2018;:k601. doi:10.1136/bmj.k601

6 Jandoc R, Burden AM, Mamdani M, *et al.* Interrupted time series analysis in drug utilization research is increasing: systematic review and recommendations. *J Clin Epidemiol* 2015;**68**:950–6. doi:10.1016/j.jclinepi.2014.12.018

7 Bärnighausen T, Oldenburg C, Tugwell P, *et al.* Quasi-experimental study designs series—paper 7: assessing the assumptions. *J Clin Epidemiol* 2017;**89**:53–66. doi:10.1016/j.jclinepi.2017.02.017

8 Hernan MA. A definition of causal effect for epidemiological research. *J Epidemiol Community Heal* 2004;**58**:265–71. doi:10.1136/jech.2002.006361

9 Rubin DB. For objective causal inference, design trumps analysis. *Ann Appl Stat* 2008;**2**:808–40. doi:10.1214/08-AOAS187

10 Rubin DB. The designversus the analysis of observational studies for causal effects: parallels with the design of randomized trials. *Stat Med* 2007;**26**:20–36. doi:10.1002/sim.2739

11 Funk MJ, Westreich D, Wiesen C, *et al.* Doubly Robust Estimation of Causal Effects. *Am J Epidemiol* 2011;**173**:761–7. doi:10.1093/aje/kwq439

12 National Institute for Health and Care Excellence. Antisocial behaviour and conduct disorders in children and young people: recognition and management. 2013. http://guidance.nice.org.uk/CG158
