# Supplementary material for: Risk factors for disruptive behaviours: protocol for a systematic review and meta-analysis of quasi-experimental evidence
Source: BMJ Open. 2020 Sep 9;10(9):e038258. doi: 10.1136/bmjopen-2020-038258 (PMC7482491; doi:10.1136/bmjopen-2020-038258)
Supplement: Supplementary data [file bmjopen-2020-038258supp003.pdf]

Appendix C: Data Extraction Form

The below template will be used to extract data from included studies.

Inclusion/Exclusion form:

A. Reference details:

Reference details						
A1. Ref ID						
A2. 1st Author						
A3. Title of paper						
A4. Journal						
A5. Volume						
A6. Year of publication						
A7. Publication type	Paper1		Abstract2		Other3	
A8. Assessor's name	LK1		SR2		Other3	
A9. Date						

B. Study included in the review:

Yes1		No2	

Reason(s) for exclusion (if excluded):	Yes1	No2
B1. Non-human participants		
B2. Non-English language		
B3. Publication date		
B4. Study design and/or statistical analyses		
B5. Publication type		
B6. Selected on physical health problem		
B7. Selected on other developmental disorder		
B8. Selected on other mental health diagnosis		
B9. No risk factor measure		
B10. No disruptive behaviour outcome measure		
B11. Timing of measures		
B12. Duplicate (Insert Ref ID of other study)	Ref ID:	
B13. Other please specify:		

Karwatowska L, *et al. BMJ Open* 2020; 10:e038258. doi: 10.1136/bmjopen-2020-038258

Data extraction form:

Reference details						
A1. Ref ID						
A2. 1st Author						
A3. Title of paper						
A4. Journal						
A5. Volume						
A6. Year of publication						
A7. Publication type	Paper1		Abstract2		Other3	
A8. Assessor's name	LK1		SR 2		Other 3	
A9. Date						

Study details												
B1. Name of study/cohort												
B2. Design	Cross-sectional1		Prospective cohort2		Retrospective cohort3		Case-control4		Linked admin5		Other6	
B2A. If other:												
B2B. Participants birth year, if given	Average1		Max2		Min3		Other4					
B3. Country	UK1		USA2		AUS3		Other4					
B3A. If other:												

Risk factor(s) measures included				
C1. Maternal smoking during pregnancy	Yes1		No2	
C2. Harsh, coercive discipline	Yes1		No2	
C3. Maltreatment	Yes1		No2	
C4. Divorce	Yes1		No2	
C5. Maternal adolescent motherhood	Yes1		No2	
C6. Parental depression	Yes1		No2	
C7. Parental antisocial behaviour	Yes1		No2	
C8. Parental drug use	Yes1		No2	
C9. Peer deviance	Yes1		No2	

C10. Poverty	Yes1		No2	
C11. Neighbourhood disadvantage	Yes1		No2	
C12. Hyperactivity / impulsivity	Yes1		No2	
C13. Low IQ / school achievement	Yes1		No2	
C14. Drug use	Yes1		No2	
C15. Resting heart rate	Yes1		No2	
C16. Skin conductance	Yes1		No2	
C17. Autonomic fear conditioning	Yes1		No2	
C18. Maternal alcohol use in pregnancy	Yes1		No2	
C19. Parental education	Yes1		No2	
C20. Family size	Yes1		No2	
C21. Bullying / peer victimisation	Yes1		No2	
C22. Stressful / adverse life events	Yes1		No2	
C23. Other:	Yes1		No2	

For each measure of risk factor(s)										
C24. Indicate which risk factor:										
C25. How was it measured:	Prospectively1		Retrospectively2							
C26. How was it ascertained	Parent1		Self2		Teacher3		Observer4		Others5	
C27. Age(s) recorded (years)	Average1		Max2		Min3		Other4			
C28. Age recorded (groups)	Childhood1		Adolescence2		Adulthood3					
C29. Above answers the same for all risk factors	Yes1		No2							

Karwatowska L, *et al. BMJ Open* 2020; 10:e038258. doi: 10.1136/bmjopen-2020-038258

If no, copy and paste below as required										
C24. Indicate which risk factor:										
C25. Psychometric properties of measure given										
C26. How was it measured:	Prospectively1		Retrospectively2							
C27. How was it ascertained	Parent1		Self2		Teacher3		Observer4		Others5	
C28. Age(s) recorded (years)	Average1		Max2		Min3		Other4			
C29. Age recorded (groups)	Childhood1		Adolescence2		Adulthood3					

Disruptive behaviour measures included										
D1. Conduct problems	Child Behaviour Checklist (CBCL)1 *or subset of CBCL e.g. Behavior Problems Index (BPI)		Eyberg Child Behavior Inventory2		Rutter Revised Preschool Scales3		DSM diagnostic items4 *or measure that uses DSM criteria e.g. Child and Adolescent Psychiatric Assessment (CAPA) or Semi-Structured Assessment for the Genetics of Alcoholism (SSAGA)		Others5	
D1A. If other please describe										
D2. Conduct disorder	Diagnostic Interview Schedule for Children (DISC)1		Diagnostic Interview for Children and Adolescents - Revised (DICA-R)2		Schedule of Affective Disorders and Schizophrenia for School-Age Children—Present and Lifetime3		DSM diagnostic items4 *or measure that uses DSM criteria e.g. Child and Adolescent Psychiatric Assessment (CAPA) or Semi-Structured Assessment for the Genetics of Alcoholism (SSAGA)		Others5	
D2A. If other please describe										
D3. Oppositional defiant disorder	The Oppositional Defiant Disorder Rating Scale1		Behavior Assessment System for Children: Second Edition (BASC-2)2		Child Behavior Checklist (CBCL)3		DSM diagnostic items4		Others5	

D3A. If other please describe										
D4. Antisocial personality disorder	Structured Clinical Interview for DSM-IV (SCID)[1]		Psychopathy Checklist – Revised (PCL-R)[2]		Mini International Neuropsychiatric Interview (MINI)[3]		International Personality Disorder Examination (IPDE)[4]		Others[5]	
D4A. If other please describe										
D5. Other disruptive behaviour										
D6. Psychometric properties of measure given										
D7. How ascertained	Parent[1]		Self[2]		Teacher[3]		Observer[4]		Others[5]	
D7B. If Other, please describe										
D8. Age(s) ascertained (years)										
D8B. Age of onset	Years[1]		Months[2]		Childhood[3]		Adolescence[4]		Others[5]	
D9. Comordibities	Yes[1]		No[2]							
D10A. If Yes, please describe										
D11. Comments										

Available participant numbers						
E1. Baseline	Yes[1]		No[2]		If yes, number	
E2. Excluded	Yes[1]		No[2]		If yes, number	
E3. Refused	Yes[1]		No[2]		If yes, number	
E4. Lost to follow-up	Yes[1]		No[2]		If yes, number	
E5. Other losses	Yes[1]		No[2]		If yes, number	
E6. Included in analysis	Yes[1]		No[2]		If yes, number	
E7. All accounted for	Yes[1]		No[2]			
E8. Length of follow up						
E9. Procedures to deal with missing data						

Karwatowska L, *et al. BMJ Open* 2020; 10:e038258. doi: 10.1136/bmjopen-2020-038258

Variable details						
F1. Risk factor	Continuous1		Categorical2		Other3	
F1A. If other please describe						
F2. Disruptive behaviour	Continuous 1		Categorical2		Other 3	
F3. Comments						

Details of study design and analyses						
G1. Quasi-experimental features	Adoption study1		Fixed effect2		Twin study3	
	Propensity Score Matching4		Sibling study5		Regression discontinuity6	
	Instrumental variable7		Interrupted time series8		Mendelian randomisation9	
	Matching study10		Experience sample11		Ecological momentary assessment12	
	Difference in difference13		In vitro fertilisation14		Polygenic risk score15	
	Natural experiment16		Matched control17		Potential outcome18	
	Balancing covariate19		Controlled before and after20		Inverse probability weight21	
	Doubly robust regression22		Selection model or selectivity model23		Heckit model or Heckman sample selection24	
	Selection correction25		Two stage residual inclusion26		Sharp design or fuzzy design27	
	Forcing variable28		Full information maximum liklihood29		Natural control30	
G1A. If other:						
G2. Included in analysis	Males and females1		Males only2		Females only3	

G3. Model type	Linear1		Logistic2		Poisson3		Survival4		Others5	
G4. List models presented incl. unadjusted										
G5. Confounders selected	Yes1		No2							
G5A. If yes, please describe										

Effect estimates									
Association estimated and which group e.g. M/F/All		Cross sectional; longitudinal; time-to-event	No. analysed	Effect estimate given? Y/N	If N, what are the qualitative findings?	If Y, what is the type of effect estimate, category comparison/value of unit change	Effect estimate	95% CI; SE; p	Confounders adjusted for
1									
2									
3									

Potential References Identified for Inclusion		
Reference	Included in database search	Not included in database search
