# Supplementary material for: Risk factors for disruptive behaviours: protocol for a systematic review and meta-analysis of quasi-experimental evidence
Source: BMJ Open. 2020 Sep 9;10(9):e038258. doi: 10.1136/bmjopen-2020-038258 (PMC7482491; doi:10.1136/bmjopen-2020-038258)
Supplement: Supplementary data [file bmjopen-2020-038258supp004.pdf]

Appendix D: [MODIFIED] Risk Of Bias In Non-randomized Studies – of **Exposures** (ROBINS-E) assessment tool

Specify a target experiment specific to the study ____

☐ The protocol specified target experiment fully applies OR
Participant______________________
Experimental exposure______________________
Control exposure ______________________

Specify the outcome

Specify which outcome is being assessed for risk of bias (typically from among those earmarked for the Summary of Findings table). Specify whether this is a proposed benefit or harm of exposure.

____

Is your aim for this study…?

- ☐ to assess the effect of initiating intervention (as in an intention to treat analysis)
- ☐ to assess the effect of initiating and adhering to intervention (as in a per-protocol analysis)
- ☐ other (specify) ____

Specify the numerical result being assessed

In case of multiple alternative analyses being presented, specify the numeric result (e.g. RR = 1.52 (95% CI 0.83 to 2.77) and/or a reference (e.g. to a table, figure or paragraph) that uniquely defines the result being assessed.

____

Karwatowska L, *et al. BMJ Open* 2020; 10:e038258. doi: 10.1136/bmjopen-2020-038258

Preliminary consideration of confounders

Complete a row for each important confounding area (i) listed in the review protocol; and (ii) relevant to the setting of this particular study, or which the study authors identified as potentially important.

"Important" confounding areas are those for which, in the context of this study, adjustment is expected to lead to a clinically important change in the estimated effect of the exposure. "Validity" refers to whether the confounding variable or variables fully measure the area, while "reliability" refers to the precision of the measurement (more measurement error means less reliability).

<table>
<tr><th colspan="2">Confounding</th><th colspan="3"></th></tr>
<tr><td colspan="5">(i) Confounding areas listed in the review protocol</td></tr>
<tr><td>Confounding area</td><td>Measured variable(s)</td><td>Is there evidence that controlling for this variable was unnecessary?</td><td>Is the confounding area measured validly and reliably by this variable (or these variables)?</td><td>OPTIONAL: Is adjusting for this variable (alone) expected to move the effect estimate up or down?</td></tr>
<tr><td rowspan="2"></td><td></td><td></td><td rowspan="2">Yes/No/No information</td><td>Favour intervention / Favour control / No information</td></tr>
<tr><td></td><td></td><td></td></tr>
<tr><td rowspan="2"></td><td></td><td></td><td rowspan="2">Yes/No/No information</td><td>Favour intervention / Favour control / No information</td></tr>
<tr><td></td><td></td><td></td></tr>
<tr><td colspan="5">(ii) Additional confounding areas relevant to the setting of this particular study, or which the study authors identified as important</td></tr>
</table>

Karwatowska L, *et al. BMJ Open* 2020; 10:e038258. doi: 10.1136/bmjopen-2020-038258

**Confounding area**	**Measured variable(s)**	**Is there evidence that controlling for this variable was unnecessary?**	**Is the confounding area measured validly and reliably by this variable (or these variables)?**	**OPTIONAL: Is adjusting for this variable (alone) expected to move the effect estimate up or down?**
			Yes/No/No information	Favour intervention / Favour control / No information
			Yes/No/No information	Favour intervention / Favour control / No information

* In the context of a particular study, variables can be demonstrated not to be confounders and so not included in the analysis: (a) if they are not predictive of the outcome; (b) if they are not predictive of exposure; or (c) because adjustment makes no or minimal difference to the estimated effect of the primary parameter. Note that “no statistically significant association” is not the same as “not predictive”.

Preliminary consideration of criteria used to determine the accuracy of measurement of exposure and outcome

Complete a row for each measure listed in the study for the (i) exposure and (ii) outcome. Of the measures listed in the protocol, consider the sensitivity, specificity, and confidence in the methods used in the study.

**Accuracy of measurement**		
**(i) Exposure measurement method listed in the study**		
**Method of measurement**	**Measured exposure**	**Is the exposure measured validly and reliably by this method (or these methods)?**
		Yes/No/No information
**(ii) Outcome measurement method listed in the study**		
**Method of measurement**	**Measured exposure**	**Is the exposure measured validly and reliably by this method (or these methods)?**
		Yes/No/No information

Preliminary consideration of co-exposures

Complete a row for each important co-intervention (i) listed in the review protocol; and (ii) relevant to the setting of this particular study, or which the study authors identified as important.

"Important" co-interventions are those for which, in the context of this study, adjustment is expected to lead to a clinically important change in the estimated effect of the intervention.

**Co-exposures**		
**(i) Co-exposures listed in the review protocol**		
**Co-exposure**	**Is there evidence that controlling for this co-exposure was unnecessary (e.g., because it was not administered)?**	**Is presence of this co-exposure likely to favor outcomes in the experimental or the control group**
		Favor experimental / Favor comparator / No information
		Favor experimental / Favor comparator / No information
		Favor experimental / Favor comparator / No information
(ii) **Additional co-exposures relevant to the setting of this particular study, or which the study authors identified as important**		
**Co-exposure**	**Is there evidence that controlling for this co-exposure was unnecessary (e.g., because it was not administered)?**	**Is presence of this co-exposure likely to favor outcomes in the experimental or the control group**
		Favor experimental / Favor comparator / No information
		Favor experimental / Favor comparator / No information
		Favor experimental / Favor comparator / No information

Karwatowska L, *et al. BMJ Open* 2020; 10:e038258. doi: 10.1136/bmjopen-2020-038258

Risk of bias assessment (cohort type studies)

Subsections	
1	Confounding
2	Selection of participants
3	Classification of exposure status
4	Departure from intended exposures
5	Missing Data
6	Measurement of outcomes
7	Selection of reported results
8	Overall judgement

Key	
NA	Not applicable
Y	Yes
PY	Potentially yes
PN	Potentially no
N	No
NI	No information

Confounding

**Bias due to confounding**		
**Question**	**Description**	**Response options**
1.1 Is there potential for confounding of the effect of exposure in this study?<br><br>If N or PN to 1.1: the study can be considered to be at low risk of bias due to confounding and no further signalling questions need be considered		Y / PY / PN / N
1.2 If Y or PY to 1.1: Was the analysis based on splitting follow up time according to exposure received?		NA / Y / PY / PN / N / NI

Karwatowska L, *et al. BMJ Open* 2020; 10:e038258. doi: 10.1136/bmjopen-2020-038258

If N or PN to 1.2, answer questions 1.4 to 1.6, which relate to baseline confounding		
1.3. **If Y or PY to 1.2**: Were exposure discontinuations or switches likely to be related to factors that are prognostic for the outcome?		NA / Y / PY / PN / N / NI
**If N or PN to 1.3**, answer questions 1.4 to 1.6, which relate to baseline confounding		
1.4. Did the authors use an appropriate analysis method that adjusted for all the critically important confounding areas?		NA / Y / PY / PN / N / NI
1.5. **If Y or PY to 1.4**: Were confounding areas that were adjusted for measured validly and reliably by the variables available in this study?		NA / Y / PY / PN / N / NI
1.6. Did the authors avoid adjusting for post-exposure variables?		NA / Y / PY / PN / N / NI
**If Y or PY to 1.3**, answer questions 1.7 and 1.8, which relate to time-varying confounding		
1.7. Did the authors use an appropriate analysis method that adjusted for all the critically important confounding areas and for time-varying confounding?		NA / Y / PY / PN / N / NI
1.8. **If Y or PY to 1.7**: Were confounding areas that were adjusted for measured validly and reliably by the variables available in this study?		NA / Y / PY / PN / N / NI

**Judgement for bias due to confounding**		
**Risk of bias**		**Low / Moderate / Serious / Critical / NI**
Optional: What is the predicted direction of bias due to confounding?		Favours experimental / Favours comparator / Unpredictable

Karwatowska L, *et al. BMJ Open* 2020; 10:e038258. doi: 10.1136/bmjopen-2020-038258

Selection of participants

Bias in selection of participants into the study		
**Question**	**Description**	**Response options**
2.1. Was selection of participants into the study (or into the analysis) based on participant characteristics observed after exposure status were measured?<br><br>**If N or PN to 2.1 go to 2.4**		Y / PY / PN / N / NI
2.2. **If Y/PY to 2.1:** Were the post-exposure variables that influenced selection associated with exposure?	Do not understand?	Y / PY / PN / N / NI
2.3. **If Y/PY to 2.2:** Were the post-exposure variables that influenced eligibility selection influenced by the outcome or a cause of the outcome?	Do not understand?	NA / Y / PY / PN / N / NI
2.4 Do start of follow-up and start of exposure coincide for most participants?		NA / Y / PY / PN / N / NI
2.5 If Y/PY to 2.2 and 2.3, or N/PN to 2.4: Were adjustment techniques used that are likely to correct for the presence of selection biases?	IVPW<br>MCS	NA / Y / PY / PN / N / NI

Judgement for selection of participants		
**Risk of bias**		**Low / Moderate / Serious / Critical / NI**
Optional: What is the predicted direction of bias due to selection of participants into the study?		Favors experimental / Favors comparator / Towards null /Away from null / Unpredictable

Classification of exposures

Bias in classification of exposures		
**Question**	**Description**	**Response options**
3.1 Is exposure status well defined?		Y / PY / PN / N / NI
3.2 Did entry into the study begin with start of the exposure?		Y / PY / PN / N / NI
3.3 Was information used to define exposure status recorded prior to outcome assessment?	Misclassification of exposure because you know the outcome [practical/actual bias]	Y / PY / PN / N / NI
3.4 Could classification of exposure status have been affected by knowledge of the outcome or risk of the outcome?	[potential bias]	Y / PY / PN / N / NI
3.5 Were exposure assessment methods robust (including methods used to input data)?		Y / PY / PN / N / NI

Judgement for classification of exposure		
**Risk of bias**		**Low / Moderate / Serious / Critical / NI**
Optional: What is the predicted direction of bias due to measurement of outcomes or exposures?		Favors experimental / Favors comparator / Towards null /Away from null / Unpredictable

Bias due to departures from intended exposures

**Bias in departures from intended exposures**		
**Question**	**Description**	**Response options**
4.1. Is there concern that changes in exposure status occurred among participants?<br><br>**If your aim for this study is to assess the effect of initiating and adhering to an exposure (as in a per-protocol analysis), answer questions 4.2 and 4.3, otherwise continue to 4.4 if Y or PY to 4.1.**		Y / PY / PN / N / NI
4.2. Did many participants switch to other exposures?		Y / PY / PN / N / NI
4.3. Were the critical co-exposures balanced across exposure groups?	If the exposure is time varying, did the authors target the effect of the initial exposure or the sustained exposure.	Y / PY / PN / N / NI
4.4. **If NY/PN PY to 4.1, or Y/PY to 4.2, or 4.3:** Were adjustment techniques used that are likely to correct for these issues?	Sustained treatment = focus; what methods were used?	NA / Y / PY / PN / N / NI

**Judgement for classification of exposure**		
**Risk of bias**		**Low / Moderate / Serious / Critical / NI**
Optional: What is the predicted direction of bias due to departures from the intended exposures?		Favors experimental / Favors comparator / Towards null

Karwatowska L, *et al. BMJ Open* 2020; 10:e038258. doi: 10.1136/bmjopen-2020-038258

Missing data

Bias due to missing data		
**Question**	**Description**	**Response options**
5.1 Were there missing outcome data?		Y / PY / PN / N / NI
5.2 Were participants excluded due to missing data on exposure status?		Y / PY / PN / N / NI
5.3 Were participants excluded due to missing data on other variables needed for the analysis?		Y / PY / PN / N / NI
5.4 **If Y/PY to 5.1, 5.2 or 5.3:** Are the proportion of participants and reasons for missing data similar across exposures?		NA / Y / PY / PN / N / NI
5.5 **If Y/PY to 5.1, 5.2 or 5.3:** Were appropriate statistical methods used to account for missing data?		NA / Y / PY / PN / N / NI

Judgement for missing data		
**Risk of bias**		**Low / Moderate / Serious / Critical / NI**
Optional: What is the predicted direction of bias due to missing data?		Favors experimental / Favors comparator / Towards null /Away from null / Unpredictable

Karwatowska L, *et al. BMJ Open* 2020; 10:e038258. doi: 10.1136/bmjopen-2020-038258

Measurement of outcomes

Bias in measurement of outcomes		
**Question**	**Description**	**Response options**
6.1 Could the outcome measure have been influenced by knowledge of the exposure received?		Y / PY / PN / N / NI
6.2 Was the outcome measure sensitive?	Sensitivity and specificity of the measurement<br>[a) were the psychometric properties of the outcome measure given]	Y / PY / PN / N / NI
6.3 Were outcome assessors unaware of the exposure received by study participants?		Y / PY / PN / N / NI
6.4 Were the methods of outcome assessment comparable across exposure groups?		Y / PY / PN / N / NI
6.5 Were any systematic errors in measurement of the outcome unrelated to exposure received?		Y / PY / PN / N / NI

Judgement for measurements of outcomes		
**Risk of bias**		**Low / Moderate / Serious / Critical / NI**
Optional: What is the predicted direction of bias due to measurement of outcomes?		Favors experimental / Favors comparator / Towards null /Away from null / Unpredictable

Karwatowska L, *et al. BMJ Open* 2020; 10:e038258. doi: 10.1136/bmjopen-2020-038258

Selection of the reported results

**Bias in selection of the reported result**		
**Question**	**Description**	**Response options**
Is the reported effect estimate likely to be selected, on the basis of the results, from...		
7.1. ... multiple outcome *measurements* within the outcome domain?	If you have multiple outcome measurements, only picking those which are significant	Y / PY / PN / N / NI
7.2 ... multiple *analyses* of the exposure-outcome relationship?	If you have run multiple analyses, only picking those which are significant	Y / PY / PN / N / NI
7.3 ... different *subgroups*?	If you have many different subgroups (e.g. males vs females), only picking those which are signficant	Y / PY / PN / N / NI

**Judgement for selection of the reported results**		
**Risk of bias**		**Low / Moderate / Serious / Critical / NI**
Optional: What is the predicted direction of bias due to selection of the reported result?		Favors experimental / Favors comparator / Towards null /Away from null / Unpredictable

Overall judgement

**Overall judgement**		
**Risk of bias**		**Low / Moderate / Serious / Critical / NI**
Optional: What is the overall predicted direction of bias for this outcome?		Favors experimental / Favors comparator / Towards null /Away from null / Unpredictable
