# Supplementary material for: Risk factors for disruptive behaviours: protocol for a systematic review and meta-analysis of quasi-experimental evidence
Source: BMJ Open. 2020 Sep 9;10(9):e038258. doi: 10.1136/bmjopen-2020-038258 (PMC7482491; doi:10.1136/bmjopen-2020-038258)
Supplement: Author's manuscript [file bmjopen-2020-038258.draft_revisions.pdf]

BMJ Open

BMJ Open is committed to open peer review. As part of this commitment we make the peer review history of every article we publish publicly available.

When an article is published we post the peer reviewers' comments and the authors' responses online. We also post the versions of the paper that were used during peer review. These are the versions that the peer review comments apply to.

The versions of the paper that follow are the versions that were submitted during the peer review process. They are not the versions of record or the final published versions. They should not be cited or distributed as the published version of this manuscript.

BMJ Open is an open access journal and the full, final, typeset and author-corrected version of record of the manuscript is available on our site with no access controls, subscription charges or pay-per-view fees (http://bmjopen.bmj.com).

If you have any questions on BMJ Open's open peer review process please email info.bmjopen@bmj.com

Risk factors for disruptive behaviours: protocol for a systematic review and meta-analysis of quasi-experimental evidence.

Journal:	*BMJ Open*
Manuscript ID	bmjopen-2020-038258
Article Type:	Protocol
Date Submitted by the Author:	04-Mar-2020
Complete List of Authors:	Karwatowska, Lucy; University College London<br>Russell, Simon; University College London<br>Solmi, Francesca; University College London, Division of Psychiatry<br>De Stavola, Bianca ; University College London Great Ormond Street Institute of Child Health Library,<br>Jaffee, Sara; University of Pennsylvania<br>Pingault, Jean-Baptiste ; University College London<br>Viding, Essi; University College London
Keywords:	Child & adolescent psychiatry < PSYCHIATRY, Personality disorders < PSYCHIATRY, STATISTICS & RESEARCH METHODS, EPIDEMIOLOGY

SCHOLARONE™
Manuscripts

I, the Submitting Author has the right to grant and does grant on behalf of all authors of the Work (as defined in the below author licence), an exclusive licence and/or a non-exclusive licence for contributions from authors who are: i) UK Crown employees; ii) where BMJ has agreed a CC-BY licence shall apply, and/or iii) in accordance with the terms applicable for US Federal Government officers or employees acting as part of their official duties; on a worldwide, perpetual, irrevocable, royalty-free basis to BMJ Publishing Group Ltd ("BMJ") its licensees and where the relevant Journal is co-owned by BMJ to the co-owners of the Journal, to publish the Work in this journal and any other BMJ products and to exploit all rights, as set out in our licence.

The Submitting Author accepts and understands that any supply made under these terms is made by BMJ to the Submitting Author unless you are acting as an employee on behalf of your employer or a postgraduate student of an affiliated institution which is paying any applicable article publishing charge ("APC") for Open Access articles. Where the Submitting Author wishes to make the Work available on an Open Access basis (and intends to pay the relevant APC), the terms of reuse of such Open Access shall be governed by a Creative Commons licence – details of these licences and which Creative Commons licence will apply to this Work are set out in our licence referred to above.

Other than as permitted in any relevant BMJ Author's Self Archiving Policies, I confirm this Work has not been accepted for publication elsewhere, is not being considered for publication elsewhere and does not duplicate material already published. I confirm all authors consent to publication of this Work and authorise the granting of this licence.

For peer review only

Full Title: Risk factors for disruptive behaviours: protocol for a systematic review and meta-analysis of quasi-experimental evidence.

Lucy Karwatowska[1*], Simon Russell[1], Francesca Solmi[2], Bianca L De Stavola[1], Sara R Jaffee[3], Jean-Baptiste Pingault[4,5] and Essi Viding[4]

Author Affiliations:

[1] Great Ormond Street Institute of Child Health, University College London, London, UK.

[2] Division of Psychiatry, University College London, London, UK.

3 Department of Psychology, University of Pennsylvania, Philadelphia, PA, USA.

[4] Department of Clinical, Educational and Health Psychology, University College London, London, UK.

[5] Social, Genetic, and Developmental Psychiatry, King's College London, De Crespigny Park, London, UK.

* Corresponding author:

Lucy Karwatowska

Email: lucy.karwatowska.18@ucl.ac.uk

Address: Great Ormond Street Institute of Child Health,

30 Guilford Street,

London, WC1N 1EH,

UK

Author Emails:

Lucy Karwatowska: lucy.karwatowska.18@ucl.ac.uk

Simon Russell: s.russell@ucl.ac.uk

Francesca Solmi: francesca.solmi@ucl.ac.uk

Bianca L De Stavola: b.destavola@ucl.ac.uk

Sara R Jaffee: srjaffee@psych.upenn.edu

Jean-Baptiste Pingault: j.pingault@ucl.ac.uk

Essi Viding: e.viding@ucl.ac.uk

For peer review only

Abstract

Introduction: Disruptive behaviour disorders, including oppositional defiant disorder and conduct disorder, are a common set of diagnoses in childhood and adolescence, with global estimates of 5.7%, 3.6% and 2.1% for any disruptive disorder, oppositional defiant disorder and conduct disorder respectively. There are high economic and social costs associated with disruptive behaviours and the prevalence of these disorders has increased in recent years. As such, disruptive behaviours represent an escalating major public health concern and it is important to understand what factors may influence the risk of these behaviours. Such research would inform interventions that aim to prevent the development of disruptive behaviours. The current review will identify the most stringent evidence of putative risk factors for disruptive behaviour from quasi-experimental studies, which enable stronger causal inference.

Methods and analysis: The review will be carried out according to PRISMA guidelines. An electronic search of MEDLINE, Embase, PsycINFO and Web of Science Core Collection will be conducted. Initial abstract and title screening will be completed independently by two reviewers using EPPI-Reviewer 4 software. Two reviewers will also independently complete full text screening and data extraction, including risk of bias. Quasi-experimental studies in the English language which examine the association between any putative risk factor and disruptive behaviour will be included if they use a recognised measure of the risk factor and a recognised measure of disruptive behaviour (i.e. conduct disorder, conduct problems, oppositional defiant disorder and antisocial personality disorder). We will conduct a meta-analysis if we can pool a minimum of three similar studies with the same or similar exposures and outcomes.

Ethics and dissemination: The proposed review does not require ethical approval. The results will help to identify risk factors for which there is strong evidence of causal effects on disruptive behaviours and also highlight potential risk factors that require further research. The findings will be disseminated via publication in a peer-reviewed scientific journal and through presentations at international meetings and conferences.

PROSPERO registration number: CRD42020169313

For peer review only

For peer review only - http://bmjopen.bmj.com/site/about/guidelines.xhtml

Keywords: disruptive behaviour; conduct problems; conduct disorder; oppositional defiant disorder; antisocial personality disorder; quasi-experimental; causal; risk factors; meta-analysis; systematic review.

Article Summary

Strengths and limitations of this study:

- The strengths of the current study include systematically evaluating quasi-experimental research, which aims to estimate causal effects, of a wide range of risk factors for a variety of disruptive behaviours.
- The risk factors will not be selected *a priori* allowing any risk factor to be included in the review and the quality of evidence will be assessed to provide confidence in the effect estimates.
- The present study will be the first to synthesise quasi-experimental studies for disruptive behaviours and will inform future research of the most probable risk factors associated with the development of these disorders.
- The current review will focus on quasi-experimental, as opposed to experimental, evidence for risk factors. Although quasi-experimental studies are more robust than other non-experimental methods they are not immune to bias.
- Articles will only be included if they are written in English, which may introduce bias. However, this bias will be minimised by including studies that are conducted in any country as long as the full article is translated into English.

Word Count: 2608

(excluding title page, abstract, references, figures, tables or acknowledgements)

For peer review only

For peer review only - http://bmjopen.bmj.com/site/about/guidelines.xhtml

Background

Individuals who display disruptive behaviours in childhood (including conduct problems, conduct disorder and oppositional defiant disorder) engage in a range of repetitive and troublesome behaviours, such as lying, fighting and stealing. Disruptive behaviour disorders are estimated to affect 5.7% of children and adolescents globally [1], with prevalence estimates increasing in recent years [2,3]. Disruptive behaviours place a considerable economic, social and emotional burden on society [4,5] and are therefore a major public health concern. There has been a long-standing interest in understanding what factors increase (or decrease) the risk of these behaviours. Despite the large numbers of studies that have investigated this question, much of the existing evidence draws upon research that has used unrepresentative samples (i.e. high-risk or clinical samples), has explored a restricted number of risk factors and/or has only focussed on identifying associations between putative risk factors and outcomes [6,7]. In comparison, quasi-experimental studies can address most of these limitations: they often use large, representative samples, they are able to investigate risk factors for which classical randomised control trials (RCTs) are unethical, impractical or too costly, and, under certain assumptions, they can produce causal estimates [8]. Given the high societal cost of disruptive behaviours it is important that research attempts to identify the causal, as opposed to correlational, risk factors associated with these behaviours. Such research would inform efforts to design more effective interventions that aim to prevent the development of disruptive behaviour and its associated adverse long-term outcomes.

To date only one narrative review of quasi-experimental evidence for risk factors for antisocial behaviour, a common symptom of disruptive behaviour disorders, has been published [9]. The authors concluded that there was evidence that harsh parental discipline, maltreatment, parental divorce, adolescent motherhood, maternal depression, parental antisocial behaviour, peer deviance and poverty had causal effects on antisocial behaviour. However, there was no evidence of causal effects for smoking during pregnancy, paternal depression, parental alcohol use or neighbourhood disadvantage. The review was limited to eight categories of risk factors and seven types of quasi-experimental design as, at the time of writing, an insufficient number of existing studies had included other types of risk factors and/or quasi-experimental designs to warrant inclusion in the review. Therefore the review did not include many risk factors that have been proposed to have causal effects on disruptive behaviour, including father absence, domestic violence and bullying victimization. It also did

For peer review only - http://bmjopen.bmj.com/site/about/guidelines.xhtml

not include specific quasi-experimental designs, such as regression discontinuity, interrupted time series or instrumental variable analyses. However, in recent years the number of quasi-experimental studies has grown quickly and the literature on risk factors for disruptive behaviours has also increased considerably.

The aim of the current review is to identify all existing quasi-experimental studies of putative risk factors for disruptive behaviours. We will consider an inclusive range of diagnostic measures of disruptive behaviour (i.e. conduct disorder, oppositional defiant disorder and antisocial personality disorder) as opposed to general symptoms (e.g. antisocial behaviour, delinquency). Furthermore, risk factors will not be selected *a priori* thereby allowing any putative risk factor for disruptive behaviour to be included in the review. We will use a broad definition of quasi-experimental studies: whereby we consider all studies that aim to estimate causal effects between risk factors and outcomes as quasi-experimental. Hence as well as including quasi-experimental study designs (e.g. natural experiments, twin studies) we will also include studies where the propensity for exposure to a particular risk factor is controlled for by using analytical methods leading to estimates of causal effects under certain assumptions (online supplementary appendix D). Given the increase in quasi-experimental designs and the use of causal inference methods in recent years, we anticipate that there will be a sufficient number of studies to perform a meta-analysis. By systematically combining and summarising all relevant literature, the current review will aim to:

1. Identify risk factors for disruptive behaviours from quasi-experimental designs that provide more stringent evidence of causal effects;
2. Examine whether the results from these studies indicate evidence of causal effects;
3. Establish whether the results vary by:
    i) Outcome (i.e. conduct disorder, conduct problems, oppositional defiant disorder or antisocial personality disorder);
    ii) Sex;
    iii) Study design (e.g. birth cohort, twin study etc.);
    iv) Analytical methods (e.g. Mendelian randomisation, propensity score matching etc.) and/or;
    v) Data quality (e.g. bias induced by the rater assessing the exposure while knowing the outcome).

For peer review only - http://bmjopen.bmj.com/site/about/guidelines.xhtml

Methods

The current protocol has been registered with the PROSPERO database [CRD42020169313] and has been reported using the Preferred Reporting Items for Systematic Reviews and Meta-Analyses Protocols (PRISMA-P) guidelines [10] (online supplementary appendix A). Any amendments to the protocol will be made through PROSPERO.

Definition of key terms

1. Quasi-experimental studies:

Quasi-experimental studies assess causality by aiming to replicate the counterfactual framework, also known as the potential outcomes framework. The counterfactual framework consists of comparing hypothetical scenarios whereby the *same* individual is either exposed or unexposed to a risk factor [11–13]. Quasi-experimental studies aim to reproduce this comparison either by design (e.g. natural experiments or twin studies) or by employing statistical methods (e.g. fixed effects or instrumental variable analyses). These methods fall into two broad categories according to whether they invoke the assumption of no unmeasured confounding (e.g. g-methods) or exploit the presence of instrumental variables (e.g. Mendelian randomisation).

2. Disruptive behaviours:

Many terms describing disruptive behaviours are used interchangeably in the literature. For the purposes of the current review we have operationalised the following definitions in order to distinguish between key terms (online supplementary appendix D):

i) In childhood:

1. Conduct disorders: a formal diagnosis whereby an individual displays repetitive and persistent patterns of antisocial, aggressive or defiant behaviour that amounts to significant and persistent violations of age-appropriate social expectations [14] and are diagnosable as defined by the DSM-5 [15];
2. Conduct problems: an term to describe a range of repetitive and disruptive behaviours, such as lying, fighting and stealing that do not necessarily meet the threshold for a diagnosis of conduct disorder;

For peer review only

3. Oppositional defiant disorder: a formal diagnosis whereby an individual exhibits defiant and disobedient behaviour towards others as opposed to conduct disorder, whereby behaviours violate the rights of others and/or societal expectations.

ii) In adulthood:

Antisocial personality disorder: a diagnosis which involves a life-long pattern of antisocial behaviour as well as irritability and remorselessness. In the DSM-5, a diagnosis of antisocial personality disorder involves exhibiting conduct disorder in childhood.

Eligibility criteria

Studies meeting *all* of the following criteria will be included in the review:

- The study must only include human participants, i.e. studies of non-human animals will not be included;
- The study must include at least one measure of a risk factor and at least one measure of disruptive behaviour. The risk factor must have occurred, but not necessarily have been measured, before the outcome;
- Effect sizes must be reported, or there must be enough numerical information to calculate effect sizes. Studies that do not meet this eligibility criterion but report the direction of findings will be described separately;
- The study may be conducted in any country but must be published in English. Language restriction was not only chosen due to practical reasons but also because the majority of research on disruptive behaviours has been conducted in English speaking countries [7];
- The study must be published between 1980 and 2020. Date restriction was chosen to maintain a level of consistency in the definition of disruptive behaviours (i.e. conduct problems, conduct disorder, oppositional defiant disorder and antisocial personality disorder);
- The study must use a quasi-experimental design or analyses. The online supplementary appendix D includes a table of definitions for the quasi-experimental terms that will be

For peer review only

used in the current review, which has been informed and adapted from the causal inference literature [11–23].

Studies meeting *any* of the following criteria will be excluded in the review:

- The study does not meet the above inclusion criteria;
- The study is a case report, clinical trial, editorial, letter to the editor, systematic review or meta-analysis;
- The study used populations selected on participant physical health problems (e.g. cancer, seizures, surgery, low gestational age etc.). Symptoms of disruptive behaviour, (e.g. antisocial behaviour) can be exhibited by people with physical health problems. However this is not the focus of the current review's research question;
- The study used populations selected *solely* on other diagnosed developmental disorders (e.g. language disorders, learning disorders, motor disorders, autism spectrum disorders etc.) or mental health diagnoses (e.g. schizophrenia, depression, bipolar etc.). As above, disruptive behaviour can share symptomology with other developmental and mental health disorders but these go beyond the scope of the present review.

Search strategy

An electronic search will be conducted to identify all relevant studies. Databases will be searched from January 1st 1980 until March 2020. The reference list of eligible full texts will also be screened to identify additional articles.

Databases:

The selection of electronic databases was made either due to the database being relevant for the current review's research question, e.g. PsycINFO, and/or because the database is frequently used in literature searches, e.g. MEDLINE. We included the Web of Science Core Collection database as eight articles that were included in the review conducted by Jaffee and colleagues were not available in the Ovid databases but were available in Web of Science. As such, we will systematically search the following databases:

1. Ovid:
   i) MEDLINE In-Process & Other Non-Indexed Citations and Daily;

For peer review only - http://bmjopen.bmj.com/site/about/guidelines.xhtml

ii) EMBASE;
iii) PsycINFO.

2. Web of Science Core Collection:
   i) Social Sciences Citation Index;
   ii) Arts & Humanities Citation Index;
   iii) Conference Proceedings Citation Index- Science;
   iv) Conference Proceedings Citation Index- Social Science & Humanities;
   v) Book Citation Index– Science;
   vi) Book Citation Index– Social Sciences & Humanities;
   vii) Emerging Sources Citation Index.

Search terms:

The search terms used for quasi-experimental studies were adapted from a paper by Glanville et al [24] to include genetically-informed causal inference methods, such as twin designs and Mendelian randomisation, in line with changes in recent literature. The search terms for disruptive behaviours were selected to include diagnostic terms, e.g. conduct disorder and antisocial personality disorder. We decided not to include terms for symptoms associated with disruptive behaviour, such as antisocial behaviour and delinquency, as they are not specific to disruptive behaviour. As mentioned above, these behaviours are also exhibited in other disorders, including autism spectrum disorder, which are not relevant to the current review's research aims. Table 1 displays the keywords that will be used in the current review and Table 2 shows the techniques that will be employed in the database searches.

Table 1. Search Strategy

**SEARCH TERMS**	
**Database**	**MeSH Terms**
Ovid MEDLINE	**Quasi-experimental studies** – causality/; adoption/; child, adopted/; exp twins/; twin study/; propensity score/; siblings/; interrupted time series analysis/; mendelian randomization analysis/; ecological momentary assessment/; fertilization in vitro/; controlled before-after studies/; fuzzy logic/.<br><br>**Disruptive behaviours** – conduct disorder/; "attention deficit and disruptive behavior disorders"/; antisocial personality disorder/.
Ovid EMBASE	**Quasi-experimental studies** – causality/; causal attribution/; causal modelling/; quasi experimental study/; adopted child/; adoption/; twins/; twin study/; propensity score/; sibling/; sibling relation/; instrumental variable analysis/; time series analysis/; mendelian randomization analysis/; ecological momentary assessment/; in vitro fertilization/; exogenous variable/; fuzzy logic/; fuzzy system/; maximum likelihood method/.

For peer review only - http://bmjopen.bmj.com/site/about/guidelines.xhtml

	**Disruptive behaviours** – conduct disorder/; oppositional defiant disorder/; disruptive behavior/; antisocial personality disorder/; psychopathy/.
Ovid PsycINFO	**Quasi-experimental studies** – exp causality/; exp causal analysis/; exp quasi experimental methods/; adopted children/; "adoption (child)"/; adoptive parents/; twins/; exp siblings/; exp time series/; exp ecological momentary assessment/; reproductive technology/; counterfactual thinking/; fuzzy logic/; fuzzy set theory/; exp maximum likelihood/.<br><br>**Disruptive behaviours** – exp conduct disorder/; exp oppositional defiant disorder/; exp disruptive behavior disorders/; antisocial personality disorder/; psychopathy/.
Web of Science	**Quasi-experimental studies** – Not applicable.<br><br>**Disruptive behaviours** – Not applicable.
**Free Text Search Terms**	
**Concept 1 -** Quasi-experimental studies	1. Quasi-experimental studies MeSH Terms<br>2. (causal*)<br>3. ((quasiexperiment*) or (quasi experiment*))<br>4. (adopt*)<br>5. (fixed effect*)<br>6. (twin*)<br>7. (propensity score*)<br>8. (sibling*)<br>9. (regression discontinuity)<br>10. (instrumental variable*)<br>11. (interrupted time series)<br>12. (mendelian randomi?ation)<br>13. (matching stud*)<br>14. (experience sampl*)<br>15. (ecological momentary assessment*)<br>16. ((difference* in difference*) or (difference* stud*))<br>17. (in vitro fertili?ation)<br>18. ((polygenic score*) or (polygenic risk score*))<br>19. (exogenous varia*)<br>20. (natural experiment*)<br>21. (matched control*)<br>22. (counterfactual*)<br>23. (potential outcome*)<br>24. ((balancing adj3 covariate) or (imbalance adj3 covariate) or (balanced adj3 covariate) or (imbalanced adj3 covariate))<br>25. (controlled before and after) or (controlled before after))<br>26. (inverse probability weight*)<br>27. ((doubly robust regression*) or (doubly robust estimate*))<br>28. ((selection model*) or (selectivity model*))<br>29. ((heckit model*) or (heckman sample selection*))<br>30. (selection correction*)<br>31. (two stage residual inclusion*)<br>32. ((sharp design*) or (fuzzy design*))<br>33. (forcing variable*)<br>34. (full information maximum likelihood)<br>35. (natural control*)<br>36. 1 OR 2 OR 3 OR 4 OR 5 OR 6 OR 7 OR 8 OR 9 OR 10 OR 11 OR 12 OR 13 OR 14 OR 15 OR 16 OR 17 OR 18 OR 19 OR 20 OR 21 OR 22 OR 23 OR 24 OR 25 OR 26 OR 27 OR 28 OR 29 OR 30 OR 31 OR 32 OR 33 OR 34 OR 35
**Concept 2 -**	37. Disruptive behaviours MeSH Terms<br>38. (conduct problem*)<br>39. (conduct disorder*)

For peer review only - http://bmjopen.bmj.com/site/about/guidelines.xhtml

Conduct disorders	40. (oppositional defian*)<br>41. (disruptive behavior*) or (disruptive behaviour*)<br>42. (antisocial personalit*) or (anti social personalit*)<br>43. (dissocial personalit*)<br>44. ((psychopathic) or (psychopathy) or (psychopath) or (psychopaths))<br>45. 37 OR 38 OR 39 OR 40 OR 41 OR 42 OR 43 OR 44
	46. 36 AND 45<br>47. Limit to English Language<br>48. Human not animal<br>49. Not review or meta-analysis or case-report<br>50. Limit to 1980 - Current

Table 2. Tools and Techniques for Searching Databases

Technique and Description	Command	Example
**All known synonyms and spellings of key words**		*Doubly robust regression* could also be referred to as *doubly robust estimate*
**Replace up to one character in the word – allows alternative spellings to be included.**	?	*Mendelian randomi?ation* would include *Mendelian randomisation* and *Mendelian randomization*
**Truncation command** – used to acknowledge and capture alternative endings to words.	*	*Inverse probability weight** would additionally search for *inverse probability weights* and *inverse probability weighting*
**Boolean logic operators - used to either: a) identify results with at least one of the search terms present; and, b) to combine results of different search terms.**	"OR"<br>"AND"	*Conduct problem* OR conduct disorder** would retrieve articles that have *either* terms. *Conduct problem* AND causal** would only retrieve articles that have *both* terms.
**Proximity operators - used to identify words within a specified distance of each other.**	adj3	*Balancing adj3 covariate* would identify articles whereby "balancing" and "covariate" are within three words of each other.

Study selection

Citations will be imported into EPPI-Reviewer 4 (EPPI-4) [25], a data management software. EPPI-4 includes a machine learning process which will reduce the time taken to screen titles and abstracts [26]. A data extraction tool will be created (online supplementary appendix B) and checklists for assessment of study quality will be adapted from the Risk Of Bias In Non-randomised Studies - of Interventions (ROBINS-I) [27] checklist (online supplementary appendix C). Two independent reviewers (LK and FS) will complete the initial screening of abstract and titles using EPPI-4. Any uncertainties over the inclusion/exclusion of studies will be resolved by team consultation.

For peer review only

Data extraction and quality assessment

Two independent reviewers (LK and SR) will complete data extraction and quality assessments. Any discrepancies will be resolved through discussion and any missing data will be requested from study authors. The following information will be extracted from the studies using a data extraction form: study reference, project name and country, study design (e.g. cohort study), participant information (e.g. number, ethnicity, age at measurement), main exposure and outcome measurement (e.g. measurement tool, rater), confounders, additional risk factors, additional outcomes, average effect size and other relevant quantities (e.g. estimate, standard error, sample size, exclusions, attrition). The online supplementary appendix B includes further details on the information that will be extracted.

Strategy for data synthesis

We will provide a description of the data extracted from the selected studies which will include the study designs used, participant characteristics, exposures and outcomes studied, measurement tools/raters and statistical methods. The magnitude of effects and quality of the studies will be described and critiqued, with potential avenues for further research discussed.

Depending on the amount and quality of information provided in the included studies and whether evidence on exposures and outcomes is available, we will conduct either a qualitative synthesis (i.e. a systematic review) or a quantitative synthesis (i.e. a meta-analysis). We anticipate that we will have a sufficient number of studies to perform meta-analyses leading to pooled effect sizes for the association between several risk factors and specific types of disruptive behaviour. When appropriate, we will also stratify the results by predefined categories (see below). Random-effects meta-analyses will be used to account for likely study heterogeneities, with the resulting pooled estimates reported together with measures of their dispersion. The I^2 statistic will be used to quantify heterogeneity and tau-squared (τ^2) will be used to indicate the extent of between-study variance. The Metafor package in R [28] will be used to conduct the analyses.

Analysis of subgroups or subsets

When sufficient data are available, meta-analysis can be used to assess specificity of pooled effects by examining "subgroups". Results will be examined separately based on outcome, sex, study design, analytical method, in particular level of confounder adjustment, and data quality. The inclusion of moderator terms will be decided depending on the

For peer review only - http://bmjopen.bmj.com/site/about/guidelines.xhtml

information provided by the studies included in the meta-analysis and whether there is a sufficient number of studies with information on the potential moderator to warrant further analysis of subsets.

Confidence in cumulative evidence

The quality of the evidence will be judged independently by two reviewers (LK and SR) using the Grading of Recommendations Assessment, Development and Evaluation (GRADE) scale [29]. Evidence quality assessment will be performed for each outcome. Five domains will be considered (risk of bias, consistency, directness, precision, and publication bias) and the quality will be adjudicated as high, moderate, low, or very low. If there is a discrepancy between reviewers, it will be resolved by reviewer discussion or by consultation with the team as needed.

Discussion

To our knowledge, the proposed review will be the first to systematically evaluate the existing evidence of causal effects between risk factors and disruptive behaviours. The results are expected to identify the most probable risk factors for disruptive behaviours and highlight potential risk factors that could be candidates for future research. The findings will be disseminated via publication in a peer-reviewed scientific journal and through presentations at international meetings and conferences.

List of Abbreviations

EPPI-4: EPPI-Reviewer 4

GRADE: Grading of Recommendations Assessment, Development and Evaluation

PRISMA: Preferred Reporting Items for Systematic Reviews and Meta-Analysis Protocols;

PROSPERO: International Prospective Register of Systematic Reviews;

RCT: Randomised Control Trial

Declarations

Ethics approval and consent to participate

Ethical approval is not required for this review as it will synthesise data from existing studies.

Consent for publication

Not applicable.

For peer review only

Availability of data and materials

The datasets used and/or analysed during the current study are available from the corresponding author on reasonable request.

Competing interests

The authors declare that they have no competing interests.

Funding

LK is supported by a PhD studentship from the Economic and Social Research Council and the Biotechnology and Biological Sciences Research Council (ES/P000347/1).

Author contributions

LK designed the study and developed the review questions, with guidance from BDS, JBP, EV and SJ. LK drafted and registered the protocol. All authors contributed to revising the protocol manuscript. LK and FS will independently complete the initial screen. LK and SR will independently complete the data extraction and quality assessment. LK will conduct the meta-analyses. LK will write the first manuscript draft and all authors will read and approve the final version of the manuscript. LK will be the guarantor of the review.

Acknowledgements

The UCL Great Ormond Street Institute of Child Health (UCL GOS ICH) receives a proportion of funding from the Department of Health's National Institute for Health Research Biomedical Research Centre funding scheme. As LK, SR and BDS work at UCL GOS ICH, this research has benefited from funding awarded to the NIHR Great Ormond Street Hospital Biomedical Research Centre.

For peer review only

For peer review only - http://bmjopen.bmj.com/site/about/guidelines.xhtml

References

1. Polanczyk G V., Salum GA, Sugaya LS, *et al.* Annual Research Review: A meta-analysis of the worldwide prevalence of mental disorders in children and adolescents. *J Child Psychol Psychiatry* 2015;**56**:345–65. doi:10.1111/jcpp.12381
2. Patalay P, Gage SH. Changes in millennial adolescent mental health and health-related behaviours over 10 years: a population cohort comparison study. *Int J Epidemiol* 2019;**48**:1650–64. doi:10.1093/ije/dyz006
3. Collishaw S, Maughan B, Goodman R, *et al.* Time trends in adolescent mental health. *J Child Psychol Psychiatry* 2004;**45**:1350–62. doi:10.1111/j.1469-7610.2004.00842.x
4. Heeks M, Reed S, Tafsiri M, *et al.* The Economic and Social Costs of Crime. 2018.
5. Rivenbark JG, Odgers CL, Caspi A, *et al.* The high societal costs of childhood conduct problems: evidence from administrative records up to age 38 in a longitudinal birth cohort. *J Child Psychol Psychiatry* 2018;**59**:703–10. doi:10.1111/jcpp.12850
6. Rutter M. Identifying the Environmental Causes of Disease: How Should We Decide what to Believe and when to Take Action?: Report Synopsis. 2007.
7. Fairchild G, Hawes DJ, Frick PJ, *et al.* Conduct disorder. *Nat Rev Dis Prim* 2019;**5**:43. doi:10.1038/s41572-019-0095-y
8. Bärnighausen T, Tugwell P, Røttingen J-A, *et al.* Quasi-experimental study designs series—paper 4: uses and value. *J Clin Epidemiol* 2017;**89**:21–9. doi:10.1016/j.jclinepi.2017.03.012
9. Jaffee SR, Strait LB, Odgers CL. From correlates to causes: Can quasi-experimental studies and statistical innovations bring us closer to identifying the causes of antisocial behavior? *Psychol Bull* 2012;**138**:272–95. doi:10.1037/a0026020
10. Shamseer L, Moher D, Clarke M, *et al.* Preferred reporting items for systematic review and meta-analysis protocols (PRISMA-P) 2015: elaboration and explanation. *BMJ* 2015;**349**:g7647–g7647. doi:10.1136/bmj.g7647
11. Hernan MA. A definition of causal effect for epidemiological research. *J Epidemiol Community Heal* 2004;**58**:265–71. doi:10.1136/jech.2002.006361
12. Rubin DB. For objective causal inference, design trumps analysis. *Ann Appl Stat* 2008;**2**:808–40. doi:10.1214/08-AOAS187
13. Rubin DB. The designversus the analysis of observational studies for causal effects: parallels with the design of randomized trials. *Stat Med* 2007;**26**:20–36. doi:10.1002/sim.2739
14. National Institute for Health and Care Excellence. *Antisocial Behavior and Conduct*

For peer review only

For peer review only - http://bmjopen.bmj.com/site/about/guidelines.xhtml

Disorders in Children and Young People - The Nice Guideline on Recognition Intervention and Management. 2013. doi:10.1111/cch.12074_5

15 American Psychiatric Association. Diagnostic and Statistical Manual of Mental Disorders, 5th Edition (DSM-5). *Diagnostic Stat Man Ment Disord 4th Ed TR* 2013;:280. doi:10.1176/appi.books.9780890425596.744053

16 Jandoc R, Burden AM, Mamdani M, *et al.* Interrupted time series analysis in drug utilization research is increasing: systematic review and recommendations. *J Clin Epidemiol* 2015;**68**:950–6. doi:10.1016/j.jclinepi.2014.12.018

17 Bärnighausen T, Oldenburg C, Tugwell P, *et al.* Quasi-experimental study designs series—paper 7: assessing the assumptions. *J Clin Epidemiol* 2017;**89**:53–66. doi:10.1016/j.jclinepi.2017.02.017

18 Funk MJ, Westreich D, Wiesen C, *et al.* Doubly Robust Estimation of Causal Effects. *Am J Epidemiol* 2011;**173**:761–7. doi:10.1093/aje/kwq439

19 Pingault J-B, O'Reilly PF, Schoeler T, *et al.* Using genetic data to strengthen causal inference in observational research. *Nat Rev Genet* 2018;**19**:566–80. doi:10.1038/s41576-018-0020-3

20 Rutter M. 'Natural Experiments' as a Means of Testing Causal Inferences. In: *Causality: Wiley Series in Probability and Statistics*. 2012. 253–72. doi:10.1002/9781119945710.ch18

21 Gunasekara FI, Richardson K, Carter K, *et al.* Fixed effects analysis of repeated measures data. *Int J Epidemiol* Published Online First: 2014. doi:10.1093/ije/dyt221

22 Bor J, Moscoe E, Mutevedzi P, *et al.* Regression Discontinuity Designs in Epidemiology. *Epidemiology* 2014;**25**:729–37. doi:10.1097/EDE.0000000000000138

23 Davies NM, Holmes M V., Davey Smith G. Reading Mendelian randomisation studies: a guide, glossary, and checklist for clinicians. *BMJ* 2018;:k601. doi:10.1136/bmj.k601

24 Glanville J, Eyers J, Jones AM, *et al.* Quasi-experimental study designs series—paper 8: identifying quasi-experimental studies to inform systematic reviews. *J Clin Epidemiol* 2017;**89**:67–76. doi:10.1016/j.jclinepi.2017.02.018

25 Thomas J, Brunton J, Graziosi S. EPPI-Reviewer 4: software for research synthesis. 2010.

26 Shamseer L, Moher D, Clarke M, *et al.* Preferred reporting items for systematic review and meta-analysis protocols (PRISMA-P) 2015: elaboration and explanation. *BMJ* 2015;**349**:g7647–g7647. doi:10.1136/bmj.g7647

For peer review only

For peer review only - http://bmjopen.bmj.com/site/about/guidelines.xhtml

27 Sterne JA, Hernán MA, Reeves BC, *et al.* ROBINS-I: a tool for assessing risk of bias in non-randomised studies of interventions. *BMJ* 2016;:i4919. doi:10.1136/bmj.i4919

28 Viechtbauer W. Conducting Meta-Analyses in R with the metafor Package. *J Stat Softw* 2010;**36**. doi:10.18637/jss.v036.i03

29 Atkins D, Eccles M, Flottorp S, *et al.* Systems for grading the quality of evidence and the strength of recommendations I: Critical appraisal of existing approaches The GRADE Working Group. *BMC Health Serv Res* 2004;**4**:38. doi:10.1186/1472-6963-4-38

For peer review only

For peer review only - http://bmjopen.bmj.com/site/about/guidelines.xhtml

Appendix A: PRISMA-P 2015 Checklist

PRISMA-P (Preferred Reporting Items for Systematic review and Meta-Analysis Protocols) 2015 checklist: recommended items to address in a systematic review protocol*

Section and topic	Item No	Checklist item	Information reported: Yes	Information reported: No	Line Number(s)
**ADMINISTRATIVE INFORMATION**					
Title:					
Identification	1a	Identify the report as a protocol of a systematic review	☒	☐	1
Update	1b	If the protocol is for an update of a previous systematic review, identify as such	☐	☒	N/A
Registration	2	If registered, provide the name of the registry (such as PROSPERO) and registration number	☒	☐	67
Authors:					
Contact	3a	Provide name, institutional affiliation, e-mail address of all protocol authors; provide physical mailing address of corresponding author	☒	☐	4 - 32
Contributions	3b	Describe contributions of protocol authors and identify the guarantor of the review	☒	☐	372 - 377
Amendments	4	If the protocol represents an amendment of a previously completed or published protocol, identify as such and list changes; otherwise, state plan for documenting important protocol amendments	☐	☒	N/A
Support:					
Sources	5a	Indicate sources of financial or other support for the review	☒	☐	367 - 369
Sponsor	5b	Provide name for the review funder and/or sponsor	☒	☐	367 - 369
Role of sponsor or funder	5c	Describe roles of funder(s), sponsor(s), and/or institution(s), if any, in developing the protocol	☒	☐	367 - 369
**INTRODUCTION**					
Rationale	6	Describe the rationale for the review in the context of what is already known	☒	☐	90 - 124
Objectives	7	Provide an explicit statement of the question(s) the review will address with reference to participants, interventions, comparators, and outcomes (PICO)	☒	☐	126 - 153
**METHODS**					
Eligibility criteria	8	Specify the study characteristics (such as PICO, study design, setting, time frame) and report characteristics (such as years considered, language, publication status) to be used as criteria for eligibility for the review	☒	☐	200 - 237

For peer review only

Information sources	9	Describe all intended information sources (such as electronic databases, contact with study authors, trial registers or other grey literature sources) with planned dates of coverage	☒	☐	239 - 263
Search strategy	10	Present draft of search strategy to be used for at least one electronic database, including planned limits, such that it could be repeated	☒	☐	277 – 278 (Table 1)
Study records:					
Data management	11a	Describe the mechanism(s) that will be used to manage records and data throughout the review	☒	☐	281 - 289
Selection process	11b	State the process that will be used for selecting studies (such as two independent reviewers) through each phase of the review (that is, screening, eligibility and inclusion in meta-analysis)	☒	☐	281 - 289
Data collection process	11c	Describe planned method of extracting data from reports (such as piloting forms, done independently, in duplicate), any processes for obtaining and confirming data from investigators	☒	☐	291 - 300
Data items	12	List and define all variables for which data will be sought (such as PICO items, funding sources), any pre-planned data assumptions and simplifications	☒	☐	292 - 300 Appendix B
Outcomes and prioritization	13	List and define all outcomes for which data will be sought, including prioritization of main and additional outcomes, with rationale	☒	☐	Appendix B
Risk of bias in individual studies	14	Describe anticipated methods for assessing risk of bias of individual studies, including whether this will be done at the outcome or study level, or both; state how this information will be used in data synthesis	☒	☐	284 – 286 Appendix C
Data synthesis	15a	Describe criteria under which study data will be quantitatively synthesised	☒	☐	302 - 318
	15b	If data are appropriate for quantitative synthesis, describe planned summary measures, methods of handling data and methods of combining data from studies, including any planned exploration of consistency (such as I^2, Kendall's τ)	☒	☐	314 - 318
	15c	Describe any proposed additional analyses (such as sensitivity or subgroup analyses, meta-regression)	☒	☐	320 - 327
	15d	If quantitative synthesis is not appropriate, describe the type of summary planned	☒	☐	302 – 310
Meta-bias(es)	16	Specify any planned assessment of meta-bias(es) (such as publication bias across studies, selective reporting within studies)	☒	☐	284 – 286 Appendix C
Confidence in cumulative evidence	17	Describe how the strength of the body of evidence will be assessed (such as GRADE)	☒	☐	329 - 336

*** It is strongly recommended that this checklist be read in conjunction with the PRISMA-P Explanation and Elaboration (cite when available) for important clarification on the items. Amendments to a review protocol should be tracked and dated. The copyright for PRISMA-P (including checklist) is held by the PRISMA-P Group and is distributed under a Creative Commons Attribution Licence 4.0.**

From: Shamseer L, Moher D, Clarke M, Ghersi D, Liberati A, Petticrew M, Shekelle P, Stewart L, PRISMA-P Group. Preferred reporting items for systematic review and meta-analysis protocols (PRISMA-P) 2015: elaboration and explanation. BMJ. 2015 Jan 2;349(jan02 1):g7647.

For peer review only

Appendix B: Data Extraction Form

The below template will be used to extract data from included studies.

Inclusion/Exclusion form:

A. Reference details:

Reference details						
A1. Ref ID						
A2. 1st Author						
A3. Title of paper						
A4. Journal						
A5. Volume						
A6. Year of publication						
A7. Publication type	Paper[1]		Abstract[2]		Other[3]	
A8. Assessor's name	LK[1]		SR[2]		Other[3]	
A9. Date						

B. Study included in the review:

Yes[1]		No[2]	

Reason(s) for exclusion (if excluded):	Yes[1]	No[2]
B1. Ineligible exposure		
B2. Ineligible outcome		
B3. Ineligible population		
B4. Review article		
B5. Duplicate (Insert Ref ID of other study)	Ref ID:	
B6. Not in English language		
B7. Other please specify:		

For peer review only

Data extraction form:

Reference details						
A1. Ref ID						
A2. 1st Author						
A3. Title of paper						
A4. Journal						
A5. Volume						
A6. Year of publication						
A7. Publication type	Paper[1]		Abstract[2]		Other[3]	
A8. Assessor's name	LK[1]		SR[2]		Other[3]	
A9. Date						

Study details												
B1. Name of study/cohort												
B2. Design	Cross-sectional[1]		Prospective cohort[2]		Retrospective cohort[3]		Case-control[4]		Linked admin[5]		Other[6]	
B2A. If other:												
B2B. Participants birth year, if given	Average[1]		Max[2]		Min[3]		Other[4]					
B3. Country	UK[1]		USA[2]		AUS[3]		Other[4]					
B3A. If other:												

Risk factor(s) measures included				
C1. Maternal smoking during pregnancy	Yes[1]		No[2]	
C2. Harsh, coercive discipline	Yes[1]		No[2]	
C3. Maltreatment	Yes[1]		No[2]	
C4. Divorce	Yes[1]		No[2]	
C5. Maternal adolescent motherhood	Yes[1]		No[2]	
C6. Parental depression	Yes[1]		No[2]	
C7. Parental antisocial behaviour	Yes[1]		No[2]	
C8. Parental drug use	Yes[1]		No[2]	
C9. Peer deviance	Yes[1]		No[2]	

For peer review only

C10. Poverty	Yes[1]		No[2]	
C11. Neighbourhood disadvantage	Yes[1]		No[2]	
C12. Hyperactivity / impulsivity	Yes[1]		No[2]	
C13. Low IQ / school achievement	Yes[1]		No[2]	
C14. Drug use	Yes[1]		No[2]	
C15. Resting heart rate	Yes[1]		No[2]	
C16. Skin conductance	Yes[1]		No[2]	
C17. Autonomic fear conditioning	Yes[1]		No[2]	
C18. Maternal alcohol use in pregnancy	Yes[1]		No[2]	
C19. Parental education	Yes[1]		No[2]	
C20. Family size	Yes[1]		No[2]	
C21. Bullying / peer victimisation	Yes[1]		No[2]	
C22. Stressful / adverse life events	Yes[1]		No[2]	
C23. Other:	Yes[1]		No[2]	

For each measure of risk factor(s)									
C24. Indicate which risk factor:									
C25. How was it measured:	Prospectively[1]		Retrospectively[2]						
C26. How was it ascertained	Parent[1]		Self[2]		Teacher[3]		Observer[4]		Other[5]
C27. Age(s) recorded (years)	Average[1]		Max[2]		Min[3]		Other[4]		
C28. Age recorded (groups)	Childhood[1]		Adolescence[2]		Adulthood[3]				
C29. Above answers the same for all risk factors	Yes[1]		No[2]						

For peer review only

If no, copy and paste below as required										
C24. Indicate which risk factor:										
C25. Psychometric properties of measure given										
C26. How was it measured:	Prospectively[1]		Retrospectively[2]							
C27. How was it ascertained	Parent[1]		Self[2]		Teacher[3]		Observer[4]		Other[5]	
C28. Age(s) recorded (years)	Average[1]		Max[2]		Min[3]		Other[4]			
C29. Age recorded (groups)	Childhood[1]		Adolescence[2]		Adulthood[3]					

Disruptive behaviour measures included										
D1. Conduct problems	Child Behaviour Checklist (CBCL)[1] *or subset of CBCL e.g. Behavior Problems Index (BPI)		Eyberg Child Behavior Inventory[2]		Rutter Revised Preschool Scales[3]		DSM diagnostic items[4] *or measure that uses DSM criteria e.g. Child and Adolescent Psychiatric Assessment (CAPA) or Semi-Structured Assessment for the Genetics of Alcoholism (SSAGA)		Other[5]	
D1A. If other please describe										
D2. Conduct disorder	Diagnostic Interview Schedule for Children (DISC)[1]		Diagnostic Interview for Children and Adolescents - Revised (DICA-R)[2]		Schedule of Affective Disorders and Schizophrenia for School-Age Children—Present and Lifetime[3]		DSM diagnostic items[4] *or measure that uses DSM criteria e.g. Child and Adolescent Psychiatric Assessment (CAPA) or Semi-Structured Assessment for the Genetics of Alcoholism (SSAGA)		Other[5]	
D2A. If other please describe										
D3. Oppositional defiant disorder	The Oppositional Defiant Disorder Rating Scale[1]		Behavior Assessment System for Children: Second Edition (BASC-2)[2]		Child Behavior Checklist (CBCL)[3]		DSM diagnostic items[4]		Other[5]	

For peer review only

D3A. If other please describe										
D4. Antisocial personality disorder	Structured Clinical Interview for DSM-IV (SCID)[1]		Psychopathy Checklist – Revised (PCL-R)[2]		Mini International Neuropsychiatric Interview (MINI)[3]		International Personality Disorder Examination (IPDE)[4]		Other[5]	
D4A. If other please describe										
D5. Other disruptive behaviour										
D6. Psychometric properties of measure given										
D7. How ascertained	Parent[1]		Self[2]		Teacher[3]		Observer[4]		Other[5]	
D7B. If Other, please describe										
D8. Age(s) ascertained (years)										
D9. Comordibities	Yes[1]		No[2]							
D10A. If Yes, please describe										
D11. Comments										

Available participant numbers						
E1. Baseline	Yes[1]		No[2]		If yes, number	
E2. Excluded	Yes[1]		No[2]		If yes, number	
E3. Refused	Yes[1]		No[2]		If yes, number	
E4. Lost to follow-up	Yes[1]		No[2]		If yes, number	
E5. Other losses	Yes[1]		No[2]		If yes, number	
E6. Included in analysis	Yes[1]		No[2]		If yes, number	
E7. All accounted for	Yes[1]		No[2]			
E8. Length of follow up						
E9. Procedures to deal with missing data						

For peer review only

Variable details						
F1. Risk factor	Continuous[1]		Categorical[2]		Other[3]	
F1A. If other please describe						
F2. Disruptive behaviour	Continuous [1]		Categorical[2]		Other [3]	
F3. Comments						

Details of study design and analyses						
G1. Quasi-experimental features	Adoption study[1]		Fixed effect[2]		Twin study[3]	
	Propensity Score Matching[4]		Sibling study[5]		Regression discontinuity[6]	
	Instrumental variable[7]		Interrupted time series[8]		Mendelian randomisation[9]	
	Matching study[10]		Experience sample[11]		Ecological momentary assessment[12]	
	Difference in difference[13]		In vitro fertilisation[14]		Polygenic risk score[15]	
	Natural experiment[16]		Matched control[17]		Potential outcome[18]	
	Balancing covariate[19]		Controlled before and after[20]		Inverse probability weight[21]	
	Doubly robust regression[22]		Selection model or selectivity model[23]		Heckit model or Heckman sample selection[24]	
	Selection correction[25]		Two stage residual inclusion[26]		Sharp design or fuzzy design[27]	
	Forcing variable[28]		Full information maximum liklihood[29]		Natural control[30]	
G1A. If other:						
G2. Included in analysis	Males and females[1]		Males only[2]		Females only[3]	

G3. Model type	Linear[1]		Logistic[2]		Poisson[3]		Survival[4]		Other[5]	
G4. List models presented incl. unadjusted										
G5. Confounders selected	Yes[1]		No[2]							
G5A. If yes, please describe										

Effect estimates							
Association estimated and which group e.g. M/F/All		Cross sectional; longitudinal; time-to-event	No. analysed	Type of effect estimate and category comparison/value of unit change	Effect estimate	95% CI; SE; p	Confounders adjusted for
1							
2							
3							

Potential References Identified for Inclusion		
Reference	Included in database search	Not included in database search

For peer review only

Appendix C: [MODIFIED] Risk Of Bias In Non-randomized Studies – of Interventions (ROBINS-I) assessment tool

Responses underlined in green are potential markers for low risk of bias, and responses in red are potential markers for a risk of bias. Where questions relate only to sign posts to other questions, no formatting is used.

Subsections	
1	Confounding
2	Selection of participants
3	Classification of exposure status
4	Missing Data
5	Measurement of outcomes
6	Selection of reported results
7	Overall judgement

Key	
NA	Not applicable
Y	Yes
PY	Potentially yes
PN	Potentially no
N	No
NI	No information

Confounding

**Bias due to confounding**		
**Question**	**Description**	**Response options**
**Time-fixed confounding**		
1.1 Is there potential for confounding of the causal effect of the risk factor in this study?		Y / PY / PN / N
**a) If N/PN to 1.1:** the study can be considered to be at low risk of bias due to confounding and no further signalling questions for this section need to be considered		Y / PY / PN / N
**b) If Y/PY to 1.1**: determine whether there is a need to assess time-varying confounding:		Y / PY / PN / N

For peer review only - http://bmjopen.bmj.com/site/about/guidelines.xhtml

1.2. Was the analysis based on splitting participants' follow up time according to exposure status?		NA / Y / PY / PN / N / NI
**a) If N/PN**, answer questions relating to baseline confounding (1.4 to 1.6)		NA / Y / PY / PN / N / NI
**b) If Y/PY**, go to question 1.3.		NA / Y / PY / PN / N / NI
1.3. Were exposure status discontinuations or switches likely to be related to factors that are prognostic for the outcome?		NA / Y / PY / PN / N / NI
**a) If N/PN**, answer questions relating to baseline confounding (1.4 to 1.6)		NA / Y / PY / PN / N / NI
**b) If Y/PY**, answer questions relating to both baseline and time-varying confounding (1.7 and 1.8)		NA / Y / PY / PN / N / NI

**Baseline confounding only**		
**Question**	**Description**	**Response options**
1.4. Did the authors use an appropriate analysis method that controlled for all the important confounding domains?		NA / Y / PY / PN / N / NI
**a) If Y/PY to 1.4**: Were confounding domains that were controlled for measured validly and reliably by the variables available in this study?		NA / Y / PY / PN / N / NI
1.5. Did the authors control for any post-exposure variables that could have been affected by the exposure?		NA / Y / PY / PN / N / NI

For peer review only - http://bmjopen.bmj.com/site/about/guidelines.xhtml

Baseline and time-varying confounding		
**Question**	**Description**	**Response options**
1.6. Did the authors use an appropriate analysis method that controlled for all the important confounding domains and for time-varying confounding?		NA / Y / PY / PN / N / NI
**a) If Y/PY to 1.7**: Were confounding domains that were controlled for measured validly and reliably by the variables available in this study?		NA / Y / PY / PN / N / NI

Judgement for confounding		
**Risk of bias**		**Low / Moderate / Serious / Critical / NI**
Optional: What is the predicted direction of bias due to confounding?		Favours experimental / Favours comparator / Unpredictable

For peer review only - http://bmjopen.bmj.com/site/about/guidelines.xhtml

Selection of Participants

Bias in selection of participants into the study		
**Question**	**Description**	**Response options**
2.1. Was selection of participants into the study (or into the analysis) based on participant characteristics observed after exposure status were measured?		Y / PY / PN / N
**a) If N/PN to 2.1:** go to 2.3		NA / Y / PY / PN / N / NI
**b) If Y/PY to 2.1**: Were the post-exposure variables that influenced selection likely to be associated with exposure status?		NA / Y / PY / PN / N / NI
2.2 **If Y/PY to 2.1a**: Were the post-exposure variables that influenced selection likely to be influenced by the outcome or a cause of the outcome?		Y / PY / PN / N / NI
2.3. Do start of follow-up and start of exposure coincide for most participants?		NA / Y / PY / PN / N / NI
2.4. **If Y/PY to 2.1b and 2.2, or N/PN to 2.3**: Were adjustment techniques used that are likely to correct for the presence of selection biases?		NA / Y / PY / PN / N / NI

Judgement for selection of participants		
**Risk of bias**		**Low / Moderate / Serious / Critical / NI**
Optional: What is the predicted direction of bias due to selection of participants into the study?		Favours experimental / Favours comparator / Unpredictable

For peer review only - http://bmjopen.bmj.com/site/about/guidelines.xhtml

Classification of exposure status

Bias in classification of exposure status		
**Question**	**Description**	**Response options**
3.1 Were exposure groups clearly defined?		Y / PY / PN / N / NI
3.2 Was the information used to define exposure status groups recorded at the start of the study?		Y / PY / PN / N / NI
3.3 Could classification of exposure status have been affected by knowledge of the outcome or risk of the outcome?		Y / PY / PN / N / NI

Judgement for classification of exposure status		
**Risk of bias**		**Low / Moderate / Serious / Critical / NI**
Optional: What is the predicted direction of bias due to classification of exposure status?		Favours experimental / Favours comparator / Unpredictable

For peer review only

Missing data

Bias due to missing data		
**Question**	**Description**	**Response options**
5.1 Were outcome data available for all, or nearly all, participants?		Y / PY / PN / N / NI
5.2 Were participants excluded due to missing data on exposure status?		Y / PY / PN / N / NI
5.3 Were participants excluded due to missing data on other variables needed for the analysis?		Y / PY / PN / N / NI
**a) If PN/N to 5.1, or Y/PY to 5.2 or 5.3**: Are the proportion of participants and reasons for missing data similar across exposure status groups?		NA / Y / PY / PN / N / NI
**b) If PN/N to 5.1, or Y/PY to 5.2 or 5.3**: Is there evidence that results were robust to the presence of missing data?		NA / Y / PY / PN / N / NI
5.4 Were methods employed in order that all available information was used to estimate the model, e.g. full information maximum likelihood		Y / PY / PN / N / NI

Judgement for missing data		
**Risk of bias**		**Low / Moderate / Serious / Critical / NI**
Optional: What is the predicted direction of bias due to missing data?		Favours experimental / Favours comparator / Unpredictable

For peer review only

For peer review only - http://bmjopen.bmj.com/site/about/guidelines.xhtml

Measurement of outcomes

**Bias in measurement of outcomes**		
**Question**	**Description**	**Response options**
6.1 Could the outcome measure have been influenced by knowledge of the exposure status?		Y / PY / PN / N / NI
6.2 Were outcome assessors aware of the exposure status of study participants?		Y / PY / PN / N / NI
6.3 Were the methods of outcome assessment comparable across exposure status groups?		Y / PY / PN / N / NI
6.4 Were any systematic errors in measurement of the outcome related to exposure status?		Y / PY / PN / N / NI
6.5 Were known outcome measures used?		Y / PY / PN / N / NI
**a) If Y to 6.5,** were the psychometric properties of the outcome measure given?		NA / Y / PY / PN / N / NI

**Judgement for measurements of outcomes**		
**Risk of bias**		**Low / Moderate / Serious / Critical / NI**
Optional: What is the predicted direction of bias due to measurement of outcomes?		Favours experimental / Favours comparator / Unpredictable

For peer review only

Selection of the reported results

Bias in selection of the reported result		
**Question**	**Description**	**Response options**
Is the reported effect estimate likely to be selected, on the basis of the results, from...		
7.1. ... multiple outcome *measurements* within the outcome domain?		Y / PY / PN / N / NI
7.2 ... multiple *analyses* of the exposure-outcome relationship?		Y / PY / PN / N / NI
7.3 ... different *subgroups*?		Y / PY / PN / N / NI

Judgement for selection of the reported results		
**Risk of bias**		**Low / Moderate / Serious / Critical / NI**
Optional: What is the predicted direction of bias due to selection of the reported result?		Favours experimental / Favours comparator / Unpredictable

Overall judgement

Overall judgement		
**Risk of bias**		**Low / Moderate / Serious / Critical / NI**
Optional: What is the overall predicted direction of bias for this outcome?		Favours experimental / Favours comparator / Unpredictable

For peer review only

For peer review only - http://bmjopen.bmj.com/site/about/guidelines.xhtml

Appendix D: Summary of Terms

Concept 1 – Quasi-experimental terms separated into quasi-experimental study designs, quasi-experimental statistical methods and quasi-experimental features.

Term	Definition
**Quasi-experimental study designs**	
Adoption study	Adoption separates genetically related parents and children and places children in a different rearing environment. Adoption studies compare associations between exposures and outcomes in parents and their children that are genetically related (not adopted) and genetically unrelated (adopted) to them. [1]
In vitro fertilisation study	In vitro fertilization can either use either parental gametes (genetically related) or donor gametes (genetically unrelated) for fertilization. Similar to adoption studies, in vitro fertilisation studies compare associations between exposures and outcomes in parents and their children that are genetically related and genetically unrelated. [1]
Genetically informed methods	Study designs or statistical analyses that use genetic information (e.g. known genetic relationships between twins) or data on genetic variations. [1]
Natural experiment	Natural experiments use natural randomly occurring circumstances (e.g. lottery win, policy or law change) as an exposure, which is assigned "as random". [2] True natural experiments are very unusual therefore they are usually described as "quasi natural experiments" or observational studies with exogenous exposures.
Quasi-experimental study	Quasi-experimental studies assess causality by aiming to replicate the counterfactual framework, also known as the potential outcomes framework. Quasi-experimental studies aim to reproduce the counterfactual framework either by design (e.g. natural experiments or twin studies) or by employing statistical methods (e.g. propensity score matching or difference-in-difference studies). These methods fall into two broad categories according to whether they invoke the assumption of no unmeasured confounding (e.g. g-methods) or exploit the presence of instrumental variables (e.g. Mendelian randomisation).
Sibling study	On average, siblings share 50% of their segregated genetic material. Similar to twin studies, some sibling studies compare outcomes in exposed versus non-exposed siblings. [1]
Twin study	On average, dizygotic twin pairs share 50% of their segregated genes compared to monozygotic twin pairs which share 100% of their genetic material. A twin that is non-exposed to a risk factor represents a natural match to their exposed co-twin. Therefore, some twin studies compare outcomes in exposed versus non-exposed pair members. [1]
**Quasi-experimental statistical methods**	
Difference in difference study / controlled before and after study	Difference-in-Differences designs, also known as controlled before and after studies, are a type of fixed effect study. The difference before and after the exposure in the exposed group is compared to the same period of time in the non-exposed group. The exposure effect is the difference between these differences. If an exposure has a harmful effect, the outcome will occur more rapidly in individuals that receive the exposure than in individuals that do not receive the exposure. [7]

For peer review only

Fixed effects	Applied to longitudinal data with repeated measures, fixed effects methods model within-individual changes over time (i.e. variation in an individual's exposures and outcomes), as opposed to between-individual changes (i.e. variation across individuals), to remove time-invariant confounding, with each individual acting as their own control. Difference-in-difference, controlled before-and-after, experience sample and ecological momentary assessment are all examples of fixed effect analyses [3].
Instrumental variable analysis	Analyses that use variables that are associated with an exposure of interest (assumption 1), do not share any common causes with the outcome (assumption 2) and affect the outcome only through the exposure (assumption 3), also known as instrumental variables. These variables can be any traits that meet the three instrumental variable assumptions, for example genetic variants (e.g. Mendelian randomisation). [5]
Interrupted time series analysis	Interrupted time series methods use observational data collected over equally spaced intervals before and after an intervention, that is exogenous to the time series, e.g. a "natural experiment" in the real word setting. The effect of the intervention is evaluated by examining whether the data pattern (e.g. the level and slope) observed post-intervention is different to that observed pre-intervention. [6]
Regression discontinuity analysis	If treatment allocation is based on whether a patient scores below or above a predetermined cut-off value, as opposed to randomisation, then the intervention will be randomly assigned for patients close to the threshold. [4] See also "Sharp/fuzzy design" below.
**Quasi-experimental features**	
Causal effect	An exposure has a causal effect on the outcome if the outcome differs when the exposure is present compared to when the exposure is absent, all other things being equal. [8]
Counterfactual framework	The comparison of hypothetical scenarios whereby the *same* individual is either exposed or unexposed to a risk factor. Also known as the potential outcomes framework. [8–10]
Doubly robust estimation	Doubly robust estimation combines two models: outcome regression and propensity score modelling. Individually these two methods lead to unbiased estimators of the causal effect only if the respective model is correctly specified; when they are combined, through doubly robust estimation, only one of the two models needs to be correctly specified to obtain an unbiased effect estimator. [11]
Heckit model/Heckman sample selection	Similar to selection/selectivity models, these are used to handle non-ignorable missing data. Heckit/Heckman selection models assume (a) a joint distribution for the missingness and outcome processes and (b) validity in the instrument. If these assumptions are met, these models can correct bias from non-randomly selected samples.
Matching study	Researchers can attempt to create a reasonable counterfactual by accounting for confounders via matching exposed and non-exposed participants on key variables. Propensity score matching approaches can be select appropriate matches (either to cases or non-cases or both) leading to different causal effects. Quasi-experimental designs are often combined with propensity score matching approaches. [2] See "Propensity score"
Potential outcome	The outcome that would occur had the exposure been set to a particular value; see also "Counterfactual framework".

For peer review only

Propensity score	A propensity score is the probability of being exposed conditional on the confounders. It has the advantage of reducing, a potentially large, number of confounders into a scalar that contains all information that is relevant for the exposure assignment in relation to the outcome. The propensity score is used as an additional covariate in outcome regression, or as a stratifying or matching variable. Inverse probability weights derived from propensity scores also remove confounding by recreating a pseudo-sample where there is no confounding. See "Matching study".
Selection/selectivity model	A model that deals with samples that are non-randomly selected, and therefore non-representative of the target population. For example, studies affected by non-ignorable missing data.
Sharp design/Fuzzy design	These are features of regression discontinuity designs.<br><br>A sharp discontinuity regression design exploits exogenous changes to the value of an exposure /intervention to estimate its causal effect on an outcome. These changes are usually triggered by overtaking a particular (sharp) threshold in a continuous endogenous variable. Since the comparison with the threshold may be affected by random error, individuals with values near the threshold can be viewed as being "as good as" randomly allocated to the exposure and analysed as if they were in an RCT.<br><br>In a fuzzy regression discontinuity design, the threshold does not need to be defined as a sharp discontinuity as long as the probability of exposure/intervention assignment differs among those near the threshold.

Concept 2 – Disruptive behaviour terms

**Term**	**Definition**
Disruptive behaviour disorders	A range of repetitive and troublesome behaviours, such as lying, fighting and stealing. It can include one or more of the below behaviours.
Conduct disorders	A formal diagnosis whereby an individual displays repetitive and persistent patterns of antisocial, aggressive or defiant behaviour that amounts to significant and persistent violations of age-appropriate social expectations [12] and are diagnosable as defined by the DSM-5.
Conduct problems	An umbrella term to describe a range of repetitive and disruptive behaviours, such as lying, fighting and stealing that do not necessarily meet the threshold for diagnosis of conduct disorder.
Oppositional defiant disorder	A formal diagnosis whereby an individual exhibits defiant and disobedient behaviour towards others as opposed to conduct disorder, whereby behaviours violate the rights of others and/or societal expectations.
Antisocial personality disorder (DSM) /	A diagnosis which involves a life-long pattern of antisocial behaviour as well as irritability and remorselessness. By definition, a diagnosis of ASPD involves exhibiting conduct disorder in childhood.

For peer review only

dissocial personality disorder (ICD)	
Psychopathy	Psychopathy is characterised by high levels of antisocial behaviour, low levels of anxiety and high levels of attention seeking. It is a specifier for antisocial personality disorder in the DSM-5.

References

1 Pingault J-B, O'Reilly PF, Schoeler T, *et al.* Using genetic data to strengthen causal inference in observational research. *Nat Rev Genet* 2018;**19**:566–80. doi:10.1038/s41576-018-0020-3

2 Rutter M. 'Natural Experiments' as a Means of Testing Causal Inferences. In: *Causality: Wiley Series in Probability and Statistics*. 2012. 253–72. doi:10.1002/9781119945710.ch18

3 Gunasekara FI, Richardson K, Carter K, *et al.* Fixed effects analysis of repeated measures data. *Int J Epidemiol* 2014;**43**:264–9. doi:10.1093/ije/dyt221

4 Bor J, Moscoe E, Mutevedzi P, *et al.* Regression Discontinuity Designs in Epidemiology. *Epidemiology* 2014;**25**:729–37. doi:10.1097/EDE.0000000000000138

5 Davies NM, Holmes M V., Davey Smith G. Reading Mendelian randomisation studies: a guide, glossary, and checklist for clinicians. *BMJ* 2018;:k601. doi:10.1136/bmj.k601

6 Jandoc R, Burden AM, Mamdani M, *et al.* Interrupted time series analysis in drug utilization research is increasing: systematic review and recommendations. *J Clin Epidemiol* 2015;**68**:950–6. doi:10.1016/j.jclinepi.2014.12.018

7 Bärnighausen T, Oldenburg C, Tugwell P, *et al.* Quasi-experimental study designs series—paper 7: assessing the assumptions. *J Clin Epidemiol* 2017;**89**:53–66. doi:10.1016/j.jclinepi.2017.02.017

8 Hernan MA. A definition of causal effect for epidemiological research. *J Epidemiol Community Heal* 2004;**58**:265–71. doi:10.1136/jech.2002.006361

9 Rubin DB. For objective causal inference, design trumps analysis. *Ann Appl Stat* 2008;**2**:808–40. doi:10.1214/08-AOAS187

10 Rubin DB. The designversus the analysis of observational studies for causal effects: parallels with the design of randomized trials. *Stat Med* 2007;**26**:20–36. doi:10.1002/sim.2739

11 Funk MJ, Westreich D, Wiesen C, *et al.* Doubly Robust Estimation of Causal Effects. *Am J Epidemiol* 2011;**173**:761–7. doi:10.1093/aje/kwq439

12 National Institute for Health and Care Excellence. Antisocial behaviour and conduct disorders in children and young people: recognition and management. 2013. http://guidance.nice.org.uk/CG158

For peer review only

BMJ Open

Risk factors for disruptive behaviours: protocol for a systematic review and meta-analysis of quasi-experimental evidence.

Journal:	*BMJ Open*
Manuscript ID	bmjopen-2020-038258.R1
Article Type:	Protocol
Date Submitted by the Author:	08-Jun-2020
Complete List of Authors:	Karwatowska, Lucy; University College London<br>Russell, Simon; University College London<br>Solmi, Francesca; University College London, Division of Psychiatry<br>De Stavola, Bianca ; University College London Great Ormond Street Institute of Child Health Library,<br>Jaffee, Sara; University of Pennsylvania<br>Pingault, Jean-Baptiste ; University College London<br>Viding, Essi; University College London
**Primary Subject Heading**:	Mental health
Secondary Subject Heading:	Epidemiology, Research methods
Keywords:	Child & adolescent psychiatry < PSYCHIATRY, Personality disorders < PSYCHIATRY, STATISTICS & RESEARCH METHODS, EPIDEMIOLOGY

SCHOLARONE™
Manuscripts

I, the Submitting Author has the right to grant and does grant on behalf of all authors of the Work (as defined in the below author licence), an exclusive licence and/or a non-exclusive licence for contributions from authors who are: i) UK Crown employees; ii) where BMJ has agreed a CC-BY licence shall apply, and/or iii) in accordance with the terms applicable for US Federal Government officers or employees acting as part of their official duties; on a worldwide, perpetual, irrevocable, royalty-free basis to BMJ Publishing Group Ltd ("BMJ") its licensees and where the relevant Journal is co-owned by BMJ to the co-owners of the Journal, to publish the Work in this journal and any other BMJ products and to exploit all rights, as set out in our licence.

The Submitting Author accepts and understands that any supply made under these terms is made by BMJ to the Submitting Author unless you are acting as an employee on behalf of your employer or a postgraduate student of an affiliated institution which is paying any applicable article publishing charge ("APC") for Open Access articles. Where the Submitting Author wishes to make the Work available on an Open Access basis (and intends to pay the relevant APC), the terms of reuse of such Open Access shall be governed by a Creative Commons licence – details of these licences and which Creative Commons licence will apply to this Work are set out in our licence referred to above.

Other than as permitted in any relevant BMJ Author's Self Archiving Policies, I confirm this Work has not been accepted for publication elsewhere, is not being considered for publication elsewhere and does not duplicate material already published. I confirm all authors consent to publication of this Work and authorise the granting of this licence.

For peer review only

Full Title: Risk factors for disruptive behaviours: protocol for a systematic review and meta-analysis of quasi-experimental evidence.

Lucy Karwatowska[1*], Simon Russell[1], Francesca Solmi[2], Bianca L De Stavola[1], Sara R Jaffee[3], Jean-Baptiste Pingault[4,5] and Essi Viding[4]

Author Affiliations:

[1] Great Ormond Street Institute of Child Health, University College London, London, UK.

[2] Division of Psychiatry, University College London, London, UK.

3 Department of Psychology, University of Pennsylvania, Philadelphia, PA, USA.

[4] Department of Clinical, Educational and Health Psychology, University College London, London, UK.

[5] Social, Genetic, and Developmental Psychiatry, King's College London, De Crespigny Park, London, UK.

* Corresponding author:

Lucy Karwatowska
Email: lucy.karwatowska.18@ucl.ac.uk
Address: Great Ormond Street Institute of Child Health,
30 Guilford Street,
London, WC1N 1EH,
UK

Author Emails:

Lucy Karwatowska: lucy.karwatowska.18@ucl.ac.uk
Simon Russell: s.russell@ucl.ac.uk
Francesca Solmi: francesca.solmi@ucl.ac.uk
Bianca L De Stavola: b.destavola@ucl.ac.uk
Sara R Jaffee: srjaffee@psych.upenn.edu
Jean-Baptiste Pingault: j.pingault@ucl.ac.uk
Essi Viding: e.viding@ucl.ac.uk

For peer review only - http://bmjopen.bmj.com/site/about/guidelines.xhtml

Abstract

Introduction: Disruptive behaviour disorders, including oppositional defiant disorder and conduct disorder, are a common set of diagnoses in childhood and adolescence, with global estimates of 5.7%, 3.6% and 2.1% for any disruptive disorder, oppositional defiant disorder and conduct disorder respectively. There are high economic and social costs associated with disruptive behaviours and the prevalence of these disorders has increased in recent years. As such, disruptive behaviours represent an escalating major public health concern and it is important to understand what factors may influence the risk of these behaviours. Such research would inform interventions that aim to prevent the development of disruptive behaviours. The current review will identify the most stringent evidence of putative risk factors for disruptive behaviour from quasi-experimental studies, which enable stronger causal inference.

Methods and analysis: The review will be carried out according to PRISMA guidelines. An electronic search of references published between 1st January 1980 and 1st March 2020 will be conducted using MEDLINE, Embase, PsycINFO and Web of Science. Initial abstract and title screening, full-text screening and data extraction will be completed independently by two reviewers using EPPI-Reviewer 4 software. Quasi-experimental studies in the English language which examine the association between any putative risk factor and a clearly defined measure of disruptive behaviour (e.g. a validated questionnaire measure) will be included. We will conduct meta-analyses if we can pool a minimum of three similar studies with the same or similar exposures and outcomes.

Ethics and dissemination: The proposed review does not require ethical approval. The results will help to identify risk factors for which there is strong evidence of causal effects on disruptive behaviours and also highlight potential risk factors that require further research. The findings will be disseminated via publication in a peer-reviewed scientific journal and through presentations at international meetings and conferences.

PROSPERO registration number: CRD42020169313

Keywords: disruptive behaviour; conduct problems; conduct disorder; oppositional defiant disorder; antisocial personality disorder; quasi-experimental; causal; risk factors; meta-analysis; systematic review.

For peer review only

Article Summary

Strengths and limitations of this study:

- The strengths of the current study include systematically evaluating quasi-experimental research, which aims to estimate causal effects, of a wide range of risk factors for a variety of disruptive behaviours.
- The risk factors will not be selected *a priori* allowing any risk factor to be included in the review and the quality of evidence will be assessed to provide confidence in the effect estimates.
- The present study will be the first to synthesise quasi-experimental studies for disruptive behaviours and will inform future research of the most probable risk factors associated with the development of these disorders.
- The current review will focus on quasi-experimental, as opposed to experimental, methods which, despite being more robust than other non-experimental methods, are not immune to bias.
- Bias that may be introduced through solely selecting articles published in the English language will be lessened by including studies that are conducted in any country as long as the full article is translated into English.

Word Count: 2918

(excluding title page, abstract, references, figures, tables or acknowledgements)

For peer review only

For peer review only - http://bmjopen.bmj.com/site/about/guidelines.xhtml

Background

Individuals who display disruptive behaviours in childhood (including conduct problems, conduct disorder and oppositional defiant disorder) engage in a range of repetitive and troublesome behaviours, such as lying, fighting and stealing. Disruptive behaviour disorders are estimated to affect 5.7% of children and adolescents globally [1], with prevalence estimates increasing in recent years [2,3]. Disruptive behaviours place a considerable economic, social and emotional burden on society [4,5] and are therefore a major public health concern. There has been a long-standing interest in understanding what factors increase (or decrease) the risk of these behaviours. Despite the large numbers of studies that have investigated this question, much of the existing evidence draws upon research that has used unrepresentative samples (i.e. high-risk or clinical samples), has explored a restricted number of risk factors and/or has only focussed on identifying associations between putative risk factors and outcomes [6,7]. In comparison, quasi-experimental studies can address most of these limitations: they often use large, representative samples, they are able to investigate risk factors for which classical randomised control trials (RCTs) are unethical, impractical or too costly, and, under certain assumptions, they can produce causal estimates [8]. Given the high societal cost of disruptive behaviours it is important that research attempts to identify the causal, as opposed to correlational, risk factors associated with these behaviours. Such research would inform efforts to design more effective interventions that aim to prevent the development of disruptive behaviour and its associated adverse long-term outcomes.

To date only one narrative review of quasi-experimental evidence for risk factors for antisocial behaviour, a common symptom of disruptive behaviour disorders, has been published [9]. The authors concluded that there was evidence that harsh parental discipline, maltreatment, parental divorce, adolescent motherhood, maternal depression, parental antisocial behaviour, peer deviance and poverty had causal effects on antisocial behaviour. However, there was no evidence of causal effects for smoking during pregnancy, paternal depression, parental alcohol use or neighbourhood disadvantage. The review was limited to eight categories of risk factors and seven types of quasi-experimental design as, at the time of writing, an insufficient number of existing studies had included other types of risk factors and/or quasi-experimental designs to warrant inclusion in the review. Therefore the review did not include many risk factors that have been proposed to have causal effects on disruptive behaviour, including father absence, domestic violence and bullying victimization. It also did

For peer review only - http://bmjopen.bmj.com/site/about/guidelines.xhtml

not include specific quasi-experimental designs, such as regression discontinuity, interrupted time series or instrumental variable analyses. However, in recent years the number of quasi-experimental studies has grown quickly and the literature on risk factors for disruptive behaviours has also increased considerably.

The aim of the current review is to identify all existing quasi-experimental studies of putative risk factors for disruptive behaviours. We will consider an inclusive range of outcomes for disruptive behaviour including diagnostic (e.g. conduct disorder, oppositional defiant disorder and antisocial personality disorder) and continuous measures (e.g. conduct problems), as opposed to general symptoms (e.g. antisocial behaviour, delinquency). Furthermore, risk factors will not be selected *a priori* thereby allowing any putative risk factor for disruptive behaviour to be included in the review. We will use a broad definition of quasi-experimental studies: whereby we consider all studies that aim to estimate causal effects between risk factors and outcomes as quasi-experimental. Hence as well as including quasi-experimental study designs (e.g. natural experiments, twin studies) we will also include studies where the propensity for exposure to a particular risk factor is controlled for by using analytical methods leading to estimates of causal effects under certain assumptions (online supplementary appendix A). Given the increase in quasi-experimental designs and the use of causal inference methods in recent years, we anticipate that there will be a sufficient number of studies to perform meta-analyses. By systematically combining and summarising all relevant literature, the current review will aim to:

1. Identify risk factors for disruptive behaviours from quasi-experimental designs that provide more stringent evidence of causal effects;
2. Examine whether the results from these studies indicate evidence of causal effects;
3. Establish whether the results vary by:
    i) Outcome (i.e. conduct disorder, conduct problems, oppositional defiant disorder or antisocial personality disorder);
    ii) Sex;
    iii) Study design (e.g. natural experiment);
    iv) Analytical methods (e.g. g-methods) and/or;
    v) Data quality (e.g. bias induced by the rater assessing the exposure while knowing the outcome).

For peer review only - http://bmjopen.bmj.com/site/about/guidelines.xhtml

Methods

The current protocol has been registered with the PROSPERO database [CRD42020169313] and has been reported using the Preferred Reporting Items for Systematic Reviews and Meta-Analyses Protocols (PRISMA-P) guidelines [10] (online supplementary appendix B). Any amendments to the protocol will be made through PROSPERO.

Definition of key terms

1. Quasi-experimental studies:

Quasi-experimental studies assess causality by aiming to replicate the counterfactual framework, also known as the potential outcomes framework. The counterfactual framework consists of comparing hypothetical scenarios whereby the *same* individual is either exposed or unexposed to a risk factor [11–13]. Quasi-experimental studies aim to reproduce this comparison either by design (e.g. natural experiments) or by employing statistical methods (e.g. g-methods). These methods fall into two broad categories according to whether they invoke the assumption of no unmeasured confounding (e.g. g-methods) or exploit the presence of instrumental variables (e.g. Mendelian randomisation).

2. Disruptive behaviours:

Many terms describing disruptive behaviours are used interchangeably in the literature. For the purposes of the current review we have operationalised the following definitions in order to distinguish between key terms (online supplementary appendix A):

i) In childhood:

1. Conduct disorders: a formal diagnosis whereby an individual displays repetitive and persistent patterns of antisocial, aggressive or defiant behaviour that amounts to significant and persistent violations of age-appropriate social expectations [14] and are diagnosable as defined by the DSM-5 [15];
2. Conduct problems: a term to describe a range of repetitive and disruptive behaviours, such as lying, fighting and stealing that do not necessarily meet the threshold for a diagnosis of conduct disorder;

For peer review only - http://bmjopen.bmj.com/site/about/guidelines.xhtml

3. Oppositional defiant disorder: a formal diagnosis whereby an individual exhibits defiant and disobedient behaviour towards others as opposed to conduct disorder, whereby behaviours violate the rights of others and/or societal expectations.

ii) In adulthood:

Antisocial personality disorder: a diagnosis which involves a life-long pattern of antisocial behaviour as well as irritability and remorselessness. In the DSM-5, a diagnosis of antisocial personality disorder involves exhibiting conduct disorder in childhood.

Eligibility criteria

Studies meeting *all* of the following criteria will be included in the review:

- The study must only include human participants, i.e. studies of non-human animals will not be included;
- The study must include at least one clearly defined measure of a risk factor and at least one clearly defined measure of disruptive behaviour, which will ensure consistency in the definition of these variables. The risk factor must have occurred, but not necessarily have been measured, before the outcome;
- Effect sizes must be reported, or there must be enough numerical information to calculate effect sizes. Studies that do not meet this eligibility criterion but only report p values or qualitative findings (e.g. "not significant" or the direction of findings) and where additional data cannot be obtained upon request will be described separately;
- The study may be conducted in any country but must be published in English. Language restriction was not only chosen due to practical reasons but also because the majority of research on disruptive behaviours has been conducted in English speaking countries [7];
- The study must be published between 1980 and 2020. Date restriction was chosen to maintain a level of consistency in the definition of disruptive behaviours;
- The study must use a quasi-experimental design or analysis. The online supplementary appendix A includes a table of definitions for the quasi-experimental terms that will be

For peer review only

used in the current review, which has been informed and adapted from the causal inference literature [11–23].

Studies meeting *any* of the following criteria will be excluded in the review:

- The study does not meet the above inclusion criteria;
- The study is a case report, clinical trial, editorial, letter to the editor, systematic review or meta-analysis;
- The study used populations selected on participant physical health problems (e.g. cancer, seizures, surgery, low gestational age etc.). Symptoms of disruptive behaviour (e.g. antisocial behaviour) can be exhibited by people with physical health problems. However this is not the focus of the current review's research question;
- The study used populations selected *solely* on other diagnosed developmental disorders (e.g. language disorders, learning disorders, motor disorders, autism spectrum disorders etc.) or mental health diagnoses (e.g. schizophrenia, depression, bipolar etc.). As above, disruptive behaviour can share symptomology with other developmental and mental health disorders but these go beyond the scope of the present review.

Search strategy

An electronic search will be conducted to identify all relevant studies. Databases will be searched from 1st January 1980 until 1st March 2020. The reference list of eligible full texts will also be screened to identify additional articles.

Databases:

The selection of electronic databases was made either due to the database being relevant for the current review's research question, e.g. PsycINFO, and/or because the database is frequently used in literature searches, e.g. MEDLINE. We included the Web of Science Core Collection database as eight articles that were included in the review conducted by Jaffee and colleagues were not available in the Ovid databases but were available in Web of Science. As such, we will systematically search the following databases:

1. Ovid:
    i) MEDLINE In-Process & Other Non-Indexed Citations and Daily;
    ii) EMBASE;

For peer review only - http://bmjopen.bmj.com/site/about/guidelines.xhtml

iii) PsycINFO.

2. Web of Science Core Collection:

i) Social Sciences Citation Index;

ii) Arts & Humanities Citation Index;

iii) Conference Proceedings Citation Index- Science;

iv) Conference Proceedings Citation Index- Social Science & Humanities;

v) Book Citation Index– Science;

vi) Book Citation Index– Social Sciences & Humanities;

vii) Emerging Sources Citation Index.

For peer review only

Search terms:

The search terms used for quasi-experimental studies were adapted from a paper by Glanville et al [24] to include genetically-informed causal inference methods, such as twin designs and Mendelian randomisation, in line with changes in recent literature. The search terms for disruptive behaviours were selected to include diagnostic terms, e.g. conduct disorder and antisocial personality disorder. We decided not to include terms for symptoms associated with disruptive behaviour, such as antisocial behaviour and delinquency, as they are not specific to disruptive behaviour. As mentioned above, these behaviours are also exhibited in other disorders, including autism spectrum disorder, which are not relevant to the current review's research aims. Table 1 displays the keywords that will be used in the current review and Table 2 shows the techniques that will be employed in the database searches.

Table 1. Search Strategy

**SEARCH TERMS**	
**Database**	**MeSH Terms**
Ovid MEDLINE	**Quasi-experimental studies** – causality/; adoption/; child, adopted/; exp twins/; twin study/; propensity score/; siblings/; interrupted time series analysis/; mendelian randomization analysis/; ecological momentary assessment/; fertilization in vitro/; controlled before-after studies/; fuzzy logic/.<br><br>**Disruptive behaviours** – conduct disorder/; "attention deficit and disruptive behavior disorders"/; antisocial personality disorder/.
Ovid EMBASE	**Quasi-experimental studies** – causality/; causal attribution/; causal modelling/; quasi experimental study/; adopted child/; adoption/; twins/; twin study/; propensity score/; sibling/; sibling relation/; instrumental variable analysis/; time series analysis/; mendelian randomization analysis/; ecological momentary assessment/; in vitro fertilization/; exogenous variable/; fuzzy logic/; fuzzy system/; maximum likelihood method/.

For peer review only - http://bmjopen.bmj.com/site/about/guidelines.xhtml

	**Disruptive behaviours** – conduct disorder/; oppositional defiant disorder/; disruptive behavior/; antisocial personality disorder/; psychopathy/.
Ovid PsycINFO	**Quasi-experimental studies** – exp causality/; exp causal analysis/; exp quasi experimental methods/; adopted children/; "adoption (child)"/; adoptive parents/; twins/; exp siblings/; exp time series/; exp ecological momentary assessment/; reproductive technology/; counterfactual thinking/; fuzzy logic/; fuzzy set theory/; exp maximum likelihood/.<br><br>**Disruptive behaviours** – exp conduct disorder/; exp oppositional defiant disorder/; exp disruptive behavior disorders/; exp externalizing symptoms/; antisocial personality disorder/; psychopathy/.
Web of Science	**Quasi-experimental studies** – Not applicable.<br><br>**Disruptive behaviours** – Not applicable.
**Free Text Search Terms**	
**Concept 1 -** Quasi-experimental studies	1. Quasi-experimental studies MeSH Terms<br>2. (causal*)<br>3. ((quasiexperiment*) or (quasi experiment*))<br>4. (adopt*)<br>5. (fixed effect*)<br>6. (twin*)<br>7. (propensity score*)<br>8. (sibling*)<br>9. (regression discontinuity)<br>10. (instrumental variable*)<br>11. (interrupted time series)<br>12. (mendelian randomi?ation)<br>13. (matching stud*)<br>14. (experience sampl*)<br>15. (ecological momentary assessment*)<br>16. ((difference* in difference*) or (difference* stud*))<br>17. (in vitro fertili?ation)<br>18. ((polygenic score*) or (polygenic risk score*))<br>19. (exogenous varia*)<br>20. (natural experiment*)<br>21. (matched control*)<br>22. (counterfactual*)<br>23. (potential outcome*)<br>24. ((balancing adj3 covariate) or (imbalance adj3 covariate) or (balanced adj3 covariate) or (imbalanced adj3 covariate))<br>25. (controlled before and after) or (controlled before after))<br>26. (inverse probability weight*)<br>27. ((doubly robust regression*) or (doubly robust estimate*))<br>28. ((selection model*) or (selectivity model*))<br>29. ((heckit model*) or (heckman sample selection*))<br>30. (selection correction*)<br>31. (two stage residual inclusion*)<br>32. ((sharp design*) or (fuzzy design*))<br>33. (forcing variable*)<br>34. (full information maximum likelihood)<br>35. (natural control*)<br>36. 1 OR 2 OR 3 OR 4 OR 5 OR 6 OR 7 OR 8 OR 9 OR 10 OR 11 OR 12 OR 13 OR 14 OR 15 OR 16 OR 17 OR 18 OR 19 OR 20 OR 21 OR 22 OR 23 OR 24 OR 25 OR 26 OR 27 OR 28 OR 29 OR 30 OR 31 OR 32 OR 33 OR 34 OR 35
	37. Disruptive behaviours MeSH Terms<br>38. (conduct problem*)

For peer review only

**Concept 2 –** Disruptive behaviours	39. (conduct disorder*)<br>40. (oppositional defian*)<br>41. (disruptive behavior*) or (disruptive behaviour*)<br>42. (externali?ing)<br>43. (antisocial personalit*) or (anti social personalit*)<br>44. (dissocial personalit*)<br>45. ((psychopathic) or (psychopathy) or (psychopath) or (psychopaths))<br>46. 37 OR 38 OR 39 OR 40 OR 41 OR 42 OR 43 OR 44 OR 45
	47. 36 AND 46<br>48. Limit to English Language<br>49. Human not animal<br>50. Not review or meta-analysis or case-report<br>51. Limit to 1980 - Current

Table 2. Tools and Techniques for Searching Databases

Technique and Description	Command	Example
**All known synonyms and spellings of key words**		*Doubly robust regression* could also be referred to as *doubly robust estimate*
**Replace up to one character in the word – allows alternative spellings to be included.**	?	*Mendelian randomi?ation* would include *Mendelian randomisation* and *Mendelian randomization*
**Truncation command** – used to acknowledge and capture alternative endings to words.	*	*Inverse probability weight** would additionally search for *inverse probability weights* and *inverse probability weighting*
**Boolean logic operators - used to either: a) identify results with at least one of the search terms present; and, b) to combine results of different search terms.**	"OR"<br>"AND"	*Conduct problem* OR conduct disorder** would retrieve articles that have *either* terms. *Conduct problem* AND causal** would only retrieve articles that have *both* terms.
**Proximity operators - used to identify words within a specified distance of each other.**	adj3	*Balancing adj3 covariate* would identify articles whereby "balancing" and "covariate" are within three words of each other.

Study selection

Citations will be imported into EPPI-Reviewer 4 (EPPI-4) [25], a data management software. EPPI-4 includes a machine learning process which will reduce the time taken to screen titles and abstracts by prioritising unscreened articles based on the reviewers' previous screening decisions; specifically, the EPPI-4 software assesses the frequency of words in the inclusion compared to the exclusion categories. Two independent reviewers (LK and FS) will complete the initial screening of abstract and titles using EPPI-4. The consistency between reviewers' screening decisions will be checked periodically and the list of unscreened references will be refreshed, allowing the machine learning software to prioritise unscreened

For peer review only - http://bmjopen.bmj.com/site/about/guidelines.xhtml

items based on relevance denoted from include/exclude codes. Once 300 references have been excluded consecutively (i.e. no references have been included) by both reviewers, the remaining references will be categorised as exclude on title and abstract. In order to double-check that these references have been correctly categorised by the software, a random selection of 5% of the unscreened references will be checked by both reviewers for inclusion. If any of these references are categorised as include, the list of unscreened references will be refreshed and both reviewers will continue screening until 300 references have been excluded consecutively. This process will be repeated until a random selection of 5% of the unscreened references are confirmed as exclude by both reviewers. Any references that are categorised as include by both reviewers after screening on title and abstracts will have their full-texts screened for inclusion by two independent reviewers (LK and FS). Any uncertainties over the inclusion/exclusion of studies will be resolved by team consultation.

Data extraction and quality assessment

Data extraction and quality assessment will be conducted by two independent reviewers (LK and SR) on any references categorised as include after full-text screening. Any discrepancies will be resolved through discussion and any missing data will be requested from study authors. A data extraction tool will be created (online supplementary appendix C) and piloted on a random selection of ten references included after full-text screening. The data extraction tool will be reviewed and modified if necessary after group discussion. The following information will be extracted from the studies using a data extraction form: study reference, project name and country, study design (e.g. cohort study), participant information (e.g. number, ethnicity, age at measurement), main exposure and outcome measurement features (e.g. measurement tool, rater, age at measurement), confounders, additional risk factors, additional outcomes, average effect size and other relevant quantities (e.g. estimate, standard error, sample size, exclusions, attrition). The online supplementary appendix B includes further details on the information that will be extracted. Checklists for assessment of study quality will be adapted from the Risk Of Bias In Non-randomised Studies - of Exposures (ROBINS-E) checklist (online supplementary appendix D).

Strategy for data synthesis

We will provide a description of the data extracted from the selected studies which will include the study designs used, participant characteristics, exposures and outcomes studied,

For peer review only - http://bmjopen.bmj.com/site/about/guidelines.xhtml

measurement tools/raters and statistical methods. The magnitude of effects and quality of the studies will be described and critiqued, with potential avenues for further research discussed.

Depending on the amount and quality of information provided in the included studies and whether evidence on exposures and outcomes is available, we will conduct either qualitative syntheses (i.e. a systematic review) or quantitative syntheses (i.e. a meta-analysis). We anticipate that we will have a sufficient number of studies to be able to perform meta-analyses leading to pooled effect sizes for the association between risk factors and disruptive behaviour. We will only conduct meta-analyses if a minimum of three studies report effect estimates on a particular risk factor and a particular outcome that are sufficiently homogenous for their meta-analysis to lead to sensible summary estimates.

When appropriate, we will also stratify the results by predefined categories (see below). Random-effects meta-analyses will be used to account for likely study heterogeneities, with the resulting pooled estimates reported together with measures of their dispersion. The I^2 statistic will be used to quantify heterogeneity and tau-squared (τ^2) will be used to indicate the extent of between-study variance. Meta-analyses of effects for binary outcomes that are measured on different scales (e.g. odds ratios and risk ratios) will be considered only if events are rare and after appropriate transformations. The Metafor package in R [26] will be used to conduct the analyses.

Analysis of subgroups

When sufficient data are available, meta-analyses can be used to assess the specificity of pooled effects by examining whether the effects vary across pre-specified “subgroups”. Subgroups will be defined according to a variety of participant (e.g. outcome, sex and age of onset) and study characteristics (e.g. study design, analytical method, in particular level of confounder adjustment, and data quality) as follows: outcomes will be categorised as either conduct problems, conduct disorder, oppositional defiant disorder or antisocial personality disorder; sex will be categorised as male or female; age of onset will either be categorised from studies providing retrospective information on continuous (e.g. months, years) or categorical data (e.g. before 5 years, 5 – 10 years etc); study design will be grouped according to subtypes (e.g. natural experiment, twin study; online supplementary appendix D); the statistical methods used for estimation will be grouped according to the assumptions invoked for causal interpretation (e.g. no unmeasured confounding or exploration of instrumental variables; online

For peer review only - http://bmjopen.bmj.com/site/about/guidelines.xhtml

supplementary appendix D); data quality will be categorised depending on the results from the data quality assessment (see below). The direct examination of heterogeneities across subgroups will be decided depending on the information provided by the studies included in the meta-analysis.

Confidence in cumulative evidence

The quality of the evidence will be judged independently by two reviewers (LK and SR) using the Grading of Recommendations Assessment, Development and Evaluation (GRADE) scale [27], with special consideration of recent guidelines for prognostic studies which share some similarities with quasi-experimental methods [28]. Evidence quality assessment will be performed for each risk factor and for each outcome. Five domains will be considered (risk of bias, consistency, directness, precision, and publication bias) and the quality will be adjudicated as high, moderate, low, or very low. If there is a discrepancy between reviewers, it will be resolved by reviewer discussion or by consultation with the team as needed. The impact of publication bias and/or unequal reporting of quantitative evidence will be examined using funnel plots.

Discussion

To our knowledge, the proposed review will be the first to systematically evaluate the existing evidence of causal effects between risk factors and disruptive behaviours. The results are expected to identify the most probable risk factors for disruptive behaviours and highlight potential risk factors that could be candidates for future research.

Ethics and dissemination

Ethical approval is not required for this review as it will synthesise data from existing studies. A manuscript detailing the results will be submitted for publication in a peer-reviewed scientific journal and presented according to the PRISMA guidelines. The results of the review will also be disseminated through presentations at international meetings and conferences.

Patient and public involvement

No patient involvement.

List of Abbreviations

EPPI-4: EPPI-Reviewer 4

For peer review only - http://bmjopen.bmj.com/site/about/guidelines.xhtml

GRADE: Grading of Recommendations Assessment, Development and Evaluation
PRISMA: Preferred Reporting Items for Systematic Reviews and Meta-Analysis Protocols;
PROSPERO: International Prospective Register of Systematic Reviews;
RCT: Randomised Control Trial

Declarations

Consent for publication

Not applicable.

Availability of data and materials

The datasets used and/or analysed during the current study are available from the corresponding author on reasonable request.

Competing interests

The authors declare that they have no competing interests.

Funding

LK is supported by a PhD studentship from the Economic and Social Research Council and the Biotechnology and Biological Sciences Research Council (ES/P000347/1). JBP is supported by the Medical Research Foundation 2018 Emerging Leaders Prize in Adolescent Mental Health.

Author contributions

LK designed the study and developed the review questions, with guidance from BDS, JBP, EV and SJ. LK drafted and registered the protocol. All authors contributed to revising the protocol manuscript. LK and FS will independently complete the initial screen. LK and SR will independently complete the data extraction and quality assessment. LK will conduct the meta-analyses. LK will write the first manuscript draft and all authors will read and approve the final version of the manuscript. LK will be the guarantor of the review.

Acknowledgements

The UCL Great Ormond Street Institute of Child Health (UCL GOS ICH) receives a proportion of funding from the Department of Health's National Institute for Health Research Biomedical Research Centre funding scheme. As LK, SR and BDS work at UCL GOS ICH, this research

For peer review only

has benefited from funding awarded to the NIHR Great Ormond Street Hospital Biomedical Research Centre.

For peer review only

For peer review only - http://bmjopen.bmj.com/site/about/guidelines.xhtml

References

1 Polanczyk G V., Salum GA, Sugaya LS, *et al.* Annual Research Review: A meta-analysis of the worldwide prevalence of mental disorders in children and adolescents. *J Child Psychol Psychiatry* 2015;**56**:345–65. doi:10.1111/jcpp.12381

2 Patalay P, Gage SH. Changes in millennial adolescent mental health and health-related behaviours over 10 years: a population cohort comparison study. *Int J Epidemiol* 2019;**48**:1650–64. doi:10.1093/ije/dyz006

3 Collishaw S, Maughan B, Goodman R, *et al.* Time trends in adolescent mental health. *J Child Psychol Psychiatry* 2004;**45**:1350–62. doi:10.1111/j.1469-7610.2004.00842.x

4 Heeks M, Reed S, Tafsiri M, *et al.* The Economic and Social Costs of Crime. 2018.

5 Rivenbark JG, Odgers CL, Caspi A, *et al.* The high societal costs of childhood conduct problems: evidence from administrative records up to age 38 in a longitudinal birth cohort. *J Child Psychol Psychiatry* 2018;**59**:703–10. doi:10.1111/jcpp.12850

6 Rutter M. Identifying the Environmental Causes of Disease: How Should We Decide what to Believe and when to Take Action? Report Synopsis. 2007.

7 Fairchild G, Hawes DJ, Frick PJ, *et al.* Conduct disorder. *Nat Rev Dis Prim* 2019;**5**:43. doi:10.1038/s41572-019-0095-y

8 Bärnighausen T, Tugwell P, Røttingen J-A, *et al.* Quasi-experimental study designs series—paper 4: uses and value. *J Clin Epidemiol* 2017;**89**:21–9. doi:10.1016/j.jclinepi.2017.03.012

9 Jaffee SR, Strait LB, Odgers CL. From correlates to causes: Can quasi-experimental studies and statistical innovations bring us closer to identifying the causes of antisocial behavior? *Psychol Bull* 2012;**138**:272–95. doi:10.1037/a0026020

10 Shamseer L, Moher D, Clarke M, *et al.* Preferred reporting items for systematic review and meta-analysis protocols (PRISMA-P) 2015: elaboration and explanation. *BMJ* 2015;**349**:g7647–g7647. doi:10.1136/bmj.g7647

11 Hernan MA. A definition of causal effect for epidemiological research. *J Epidemiol Community Heal* 2004;**58**:265–71. doi:10.1136/jech.2002.006361

12 Rubin DB. For objective causal inference, design trumps analysis. *Ann Appl Stat* 2008;**2**:808–40. doi:10.1214/08-AOAS187

13 Rubin DB. The designversus the analysis of observational studies for causal effects: parallels with the design of randomized trials. *Stat Med* 2007;**26**:20–36. doi:10.1002/sim.2739

14 National Institute for Health and Care Excellence. Antisocial behaviour and conduct

For peer review only

For peer review only - http://bmjopen.bmj.com/site/about/guidelines.xhtml

disorders in children and young people: recognition and management. 2013. http://guidance.nice.org.uk/CG158

15 American Psychiatric Association. *Diagnostic and statistical manual of mental disorders (5th ed.)*. 2013. doi:10.1176/appi.books.9780890425596

16 Pingault J-B, O'Reilly PF, Schoeler T, *et al.* Using genetic data to strengthen causal inference in observational research. *Nat Rev Genet* 2018;**19**:566–80. doi:10.1038/s41576-018-0020-3

17 Rutter M. 'Natural Experiments' as a Means of Testing Causal Inferences. In: *Causality: Wiley Series in Probability and Statistics*. 2012. 253–72. doi:10.1002/9781119945710.ch18

18 Gunasekara FI, Richardson K, Carter K, *et al.* Fixed effects analysis of repeated measures data. *Int J Epidemiol* Published Online First: 2014. doi:10.1093/ije/dyt221

19 Bor J, Moscoe E, Mutevedzi P, *et al.* Regression discontinuity designs in epidemiology: Causal inference without randomized trials. *Epidemiology* Published Online First: 2014. doi:10.1097/EDE.0000000000000138

20 Davies NM, Holmes M V., Davey Smith G. Reading Mendelian randomisation studies: a guide, glossary, and checklist for clinicians. *BMJ* 2018;:k601. doi:10.1136/bmj.k601

21 Jandoc R, Burden AM, Mamdani M, *et al.* Interrupted time series analysis in drug utilization research is increasing: systematic review and recommendations. *J Clin Epidemiol* 2015;**68**:950–6. doi:10.1016/j.jclinepi.2014.12.018

22 Bärnighausen T, Oldenburg C, Tugwell P, *et al.* Quasi-experimental study designs series—paper 7: assessing the assumptions. *J Clin Epidemiol* 2017;**89**:53–66. doi:10.1016/j.jclinepi.2017.02.017

23 Funk MJ, Westreich D, Wiesen C, *et al.* Doubly Robust Estimation of Causal Effects. *Am J Epidemiol* 2011;**173**:761–7. doi:10.1093/aje/kwq439

24 Glanville J, Eyers J, Jones AM, *et al.* Quasi-experimental study designs series—paper 8: identifying quasi-experimental studies to inform systematic reviews. *J Clin Epidemiol* 2017;**89**:67–76. doi:10.1016/j.jclinepi.2017.02.018

25 Thomas J, Brunton J, Graziosi S. EPPI-Reviewer 4: software for research synthesis. 2010.

26 Viechtbauer W. Conducting Meta-Analyses in R with the metafor Package. *J Stat Softw* 2010;**36**. doi:10.18637/jss.v036.i03

27 Atkins D, Eccles M, Flottorp S, *et al.* Systems for grading the quality of evidence and

For peer review only

For peer review only - http://bmjopen.bmj.com/site/about/guidelines.xhtml

the strength of recommendations I: Critical appraisal of existing approaches The GRADE Working Group. *BMC Health Serv Res* 2004;**4**:38. doi:10.1186/1472-6963-4-38

28 Foroutan F, Guyatt G, Zuk V, *et al.* GRADE Guidelines 28: Use of GRADE for the assessment of evidence about prognostic factors: rating certainty in identification of groups of patients with different absolute risks. *J Clin Epidemiol* 2020;**121**:62–70. doi:10.1016/j.jclinepi.2019.12.023

For peer review only

For peer review only - http://bmjopen.bmj.com/site/about/guidelines.xhtml

Appendix A: Summary of Terms

Concept 1 – Quasi-experimental terms separated into quasi-experimental study designs, quasi-experimental statistical methods and quasi-experimental features.

Term	Definition
**Quasi-experimental study designs**	
Adoption study	Adoption separates genetically related parents and children and places children in a different rearing environment. Adoption studies compare associations between exposures and outcomes in parents and their children that are genetically related (not adopted) and genetically unrelated (adopted) to them. [1]
In vitro fertilisation study	In vitro fertilization can either use either parental gametes (genetically related) or donor gametes (genetically unrelated) for fertilization. Similar to adoption studies, in vitro fertilisation studies compare associations between exposures and outcomes in parents and their children that are genetically related and genetically unrelated. [1]
Genetically informed methods	Study designs or statistical analyses that use genetic information (e.g. known genetic relationships between twins) or data on genetic variations. [1]
Natural experiment	Natural experiments use natural randomly occurring circumstances (e.g. lottery win, policy or law change) as an exposure, which is assigned “as random”. [2] True natural experiments are very unusual therefore they are usually described as “quasi natural experiments” or observational studies with exogenous exposures.
Quasi-experimental study	Quasi-experimental studies assess causality by aiming to replicate the counterfactual framework, also known as the potential outcomes framework. Quasi-experimental studies aim to reproduce the counterfactual framework either by design (e.g. natural experiments or twin studies) or by employing statistical methods (e.g. propensity score matching or difference-in-difference studies). These methods fall into two broad categories according to whether they invoke the assumption of no unmeasured confounding (e.g. g-methods) or exploit the presence of instrumental variables (e.g. Mendelian randomisation).
Sibling study	On average, siblings share 50% of their segregated genetic material. Similar to twin studies, some sibling studies compare outcomes in exposed versus non-exposed siblings. [1]
Twin study	On average, dizygotic twin pairs share 50% of their segregated genes compared to monozygotic twin pairs which share 100% of their genetic material. A twin that is non-exposed to a risk factor represents a natural match to their exposed co-twin. Therefore, some twin studies compare outcomes in exposed versus non-exposed pair members. [1]
**Quasi-experimental statistical methods**	
Difference in difference study / controlled before and after study	Difference-in-Differences designs, also known as controlled before and after studies, are a type of fixed effect study. The difference before and after the exposure in the exposed group is compared to the same period of time in the non-exposed group. The exposure effect is the difference between these differences. If an exposure has a harmful effect, the outcome will occur more rapidly in individuals that receive the exposure than in individuals that do not receive the exposure. [7]

For peer review only

Fixed effects	Applied to longitudinal data with repeated measures, fixed effects methods model within-individual changes over time (i.e. variation in an individual's exposures and outcomes), as opposed to between-individual changes (i.e. variation across individuals), to remove time-invariant confounding, with each individual acting as their own control. Difference-in-difference, controlled before-and-after, experience sample and ecological momentary assessment are all examples of fixed effect analyses [3].
Instrumental variable analysis	Analyses that use variables that are associated with an exposure of interest (assumption 1), do not share any common causes with the outcome (assumption 2) and affect the outcome only through the exposure (assumption 3), also known as instrumental variables. These variables can be any traits that meet the three instrumental variable assumptions, for example genetic variants (e.g. Mendelian randomisation). [5]
Interrupted time series analysis	Interrupted time series methods use observational data collected over equally spaced intervals before and after an intervention, that is exogenous to the time series, e.g. a "natural experiment" in the real word setting. The effect of the intervention is evaluated by examining whether the data pattern (e.g. the level and slope) observed post-intervention is different to that observed pre-intervention. [6]
Regression discontinuity analysis	If treatment allocation is based on whether a patient scores below or above a predetermined cut-off value, as opposed to randomisation, then the intervention will be randomly assigned for patients close to the threshold. [4] See also "Sharp/fuzzy design" below.
**Quasi-experimental features**	
Causal effect	An exposure has a causal effect on the outcome if the outcome differs when the exposure is present compared to when the exposure is absent, all other things being equal. [8]
Counterfactual framework	The comparison of hypothetical scenarios whereby the *same* individual is either exposed or unexposed to a risk factor. Also known as the potential outcomes framework. [8–10]
Doubly robust estimation	Doubly robust estimation combines two models: outcome regression and propensity score modelling. Individually these two methods lead to unbiased estimators of the causal effect only if the respective model is correctly specified; when they are combined, through doubly robust estimation, only one of the two models needs to be correctly specified to obtain an unbiased effect estimator. [11]
Heckit model/Heckman sample selection	Similar to selection/selectivity models, these are used to handle non-ignorable missing data. Heckit/Heckman selection models assume (a) a joint distribution for the missingness and outcome processes and (b) validity in the instrument. If these assumptions are met, these models can correct bias from non-randomly selected samples.
Matching study	Researchers can attempt to create a reasonable counterfactual by accounting for confounders via matching exposed and non-exposed participants on key variables. Propensity score matching approaches can be select appropriate matches (either to cases or non-cases or both) leading to different causal effects. Quasi-experimental designs are often combined with propensity score matching approaches. [2] See "Propensity score"
Potential outcome	The outcome that would occur had the exposure been set to a particular value; see also "Counterfactual framework".

For peer review only

Propensity score	A propensity score is the probability of being exposed conditional on the confounders. It has the advantage of reducing, a potentially large, number of confounders into a scalar that contains all information that is relevant for the exposure assignment in relation to the outcome. The propensity score is used as an additional covariate in outcome regression, or as a stratifying or matching variable. Inverse probability weights derived from propensity scores also remove confounding by recreating a pseudo-sample where there is no confounding. See "Matching study".
Selection/selectivity model	A model that deals with samples that are non-randomly selected, and therefore non-representative of the target population. For example, studies affected by non-ignorable missing data.
Sharp design/Fuzzy design	These are features of regression discontinuity designs.<br><br>A sharp discontinuity regression design exploits exogenous changes to the value of an exposure /intervention to estimate its causal effect on an outcome. These changes are usually triggered by overtaking a particular (sharp) threshold in a continuous endogenous variable. Since the comparison with the threshold may be affected by random error, individuals with values near the threshold can be viewed as being "as good as" randomly allocated to the exposure and analysed as if they were in an RCT.<br><br>In a fuzzy regression discontinuity design, the threshold does not need to be defined as a sharp discontinuity as long as the probability of exposure/intervention assignment differs among those near the threshold.

Concept 2 – Disruptive behaviour terms

Term	Definition
Disruptive behaviour disorders / externalising disorders	A range of repetitive and troublesome behaviours, such as lying, fighting and stealing. It can include one or more of the below behaviours and the term is sometimes used interchangeably with "externalising disorders".
Antisocial personality disorder (DSM) / dissocial personality disorder (ICD)	A diagnosis which involves a life-long pattern of antisocial behaviour as well as irritability and remorselessness. By definition, a diagnosis of ASPD involves exhibiting conduct disorder in childhood.
Conduct disorders	A formal diagnosis whereby an individual displays repetitive and persistent patterns of antisocial, aggressive or defiant behaviour that amounts to significant and persistent violations of age-appropriate social expectations [12] and are diagnosable as defined by the DSM-5.
Conduct problems	An umbrella term to describe a range of repetitive and disruptive behaviours, such as lying, fighting and stealing that do not necessarily meet the threshold for diagnosis of conduct disorder.

For peer review only

Oppositional defiant disorder	A formal diagnosis whereby an individual exhibits defiant and disobedient behaviour towards others as opposed to conduct disorder, whereby behaviours violate the rights of others and/or societal expectations.
Psychopathy	Psychopathy is characterised by high levels of antisocial behaviour, low levels of anxiety and high levels of attention seeking. It is a specifier for antisocial personality disorder in the DSM-5.

References

1 Pingault J-B, O'Reilly PF, Schoeler T, *et al.* Using genetic data to strengthen causal inference in observational research. *Nat Rev Genet* 2018;**19**:566–80. doi:10.1038/s41576-018-0020-3

2 Rutter M. 'Natural Experiments' as a Means of Testing Causal Inferences. In: *Causality: Wiley Series in Probability and Statistics*. 2012. 253–72. doi:10.1002/9781119945710.ch18

3 Gunasekara FI, Richardson K, Carter K, *et al.* Fixed effects analysis of repeated measures data. *Int J Epidemiol* 2014;**43**:264–9. doi:10.1093/ije/dyt221

4 Bor J, Moscoe E, Mutevedzi P, *et al.* Regression Discontinuity Designs in Epidemiology. *Epidemiology* 2014;**25**:729–37. doi:10.1097/EDE.0000000000000138

5 Davies NM, Holmes M V., Davey Smith G. Reading Mendelian randomisation studies: a guide, glossary, and checklist for clinicians. *BMJ* 2018;:k601. doi:10.1136/bmj.k601

6 Jandoc R, Burden AM, Mamdani M, *et al.* Interrupted time series analysis in drug utilization research is increasing: systematic review and recommendations. *J Clin Epidemiol* 2015;**68**:950–6. doi:10.1016/j.jclinepi.2014.12.018

7 Bärnighausen T, Oldenburg C, Tugwell P, *et al.* Quasi-experimental study designs series—paper 7: assessing the assumptions. *J Clin Epidemiol* 2017;**89**:53–66. doi:10.1016/j.jclinepi.2017.02.017

8 Hernan MA. A definition of causal effect for epidemiological research. *J Epidemiol Community Heal* 2004;**58**:265–71. doi:10.1136/jech.2002.006361

9 Rubin DB. For objective causal inference, design trumps analysis. *Ann Appl Stat* 2008;**2**:808–40. doi:10.1214/08-AOAS187

10 Rubin DB. The designversus the analysis of observational studies for causal effects: parallels with the design of randomized trials. *Stat Med* 2007;**26**:20–36. doi:10.1002/sim.2739

11 Funk MJ, Westreich D, Wiesen C, *et al.* Doubly Robust Estimation of Causal Effects. *Am J Epidemiol* 2011;**173**:761–7. doi:10.1093/aje/kwq439

12 National Institute for Health and Care Excellence. Antisocial behaviour and conduct disorders in children and young people: recognition and management. 2013. http://guidance.nice.org.uk/CG158

For peer review only

Appendix B: PRISMA-P 2015 Checklist

PRISMA-P (Preferred Reporting Items for Systematic review and Meta-Analysis Protocols) 2015 checklist: recommended items to address in a systematic review protocol*

Section and topic	Item No	Checklist item	Information reported: Yes	Information reported: No	Line Number(s)
		**ADMINISTRATIVE INFORMATION**			
Title:					
Identification	1a	Identify the report as a protocol of a systematic review	☒	☐	1
Update	1b	If the protocol is for an update of a previous systematic review, identify as such	☐	☒	N/A
Registration	2	If registered, provide the name of the registry (such as PROSPERO) and registration number	☒	☐	64
Authors:					
Contact	3a	Provide name, institutional affiliation, e-mail address of all protocol authors; provide physical mailing address of corresponding author	☒	☐	4 - 32
Contributions	3b	Describe contributions of protocol authors and identify the guarantor of the review	☒	☐	484 – 489
Amendments	4	If the protocol represents an amendment of a previously completed or published protocol, identify as such and list changes; otherwise, state plan for documenting important protocol amendments	☐	☒	N/A
Support:					
Sources	5a	Indicate sources of financial or other support for the review	☒	☐	477 – 481
Sponsor	5b	Provide name for the review funder and/or sponsor	☒	☐	477 – 481
Role of sponsor or funder	5c	Describe roles of funder(s), sponsor(s), and/or institution(s), if any, in developing the protocol	☒	☐	491 – 500
**INTRODUCTION**					
Rationale	6	Describe the rationale for the review in the context of what is already known	☒	☐	106 – 160
Objectives	7	Provide an explicit statement of the question(s) the review will address with reference to participants, interventions, comparators, and outcomes (PICO)	☒	☐	162 – 172
**METHODS**					
Eligibility criteria	8	Specify the study characteristics (such as PICO, study design, setting, time frame) and report characteristics (such as years considered, language, publication status) to be used as criteria for eligibility for the review	☒	☐	230 – 271

For peer review only

Information sources	9	Describe all intended information sources (such as electronic databases, contact with study authors, trial registers or other grey literature sources) with planned dates of coverage	▬	▬	286 - 299
Search strategy	10	Present draft of search strategy to be used for at least one electronic database, including planned limits, such that it could be repeated	▬	▬	313 – 314 (Table 1)
Study records:					
Data management	11a	Describe the mechanism(s) that will be used to manage records and data throughout the review	▬	▬	318 – 319
Selection process	11b	State the process that will be used for selecting studies (such as two independent reviewers) through each phase of the review (that is, screening, eligibility and inclusion in meta-analysis)	▬	▬	322 – 346
Data collection process	11c	Describe planned method of extracting data from reports (such as piloting forms, done independently, in duplicate), any processes for obtaining and confirming data from investigators	▬	▬	349 – 363
Data items	12	List and define all variables for which data will be sought (such as PICO items, funding sources), any pre-planned data assumptions and simplifications	▬	▬	354 – 361 Appendix B
Outcomes and prioritization	13	List and define all outcomes for which data will be sought, including prioritization of main and additional outcomes, with rationale	▬	▬	Appendix B
Risk of bias in individual studies	14	Describe anticipated methods for assessing risk of bias of individual studies, including whether this will be done at the outcome or study level, or both; state how this information will be used in data synthesis	▬	▬	361 – 363 Appendix C
Data synthesis	15a	Describe criteria under which study data will be quantitatively synthesised	▬	▬	365 – 384
	15b	If data are appropriate for quantitative synthesis, describe planned summary measures, methods of handling data and methods of combining data from studies, including any planned exploration of consistency (such as I_2, Kendall's τ)	▬	▬	386 – 393
	15c	Describe any proposed additional analyses (such as sensitivity or subgroup analyses, meta-regression)	▬	▬	395 – 423
	15d	If quantitative synthesis is not appropriate, describe the type of summary planned	▬	▬	239 – 242
Meta-bias(es)	16	Specify any planned assessment of meta-bias(es) (such as publication bias across studies, selective reporting within studies)	▬	▬	434 – 435 Appendix C
Confidence in cumulative evidence	17	Describe how the strength of the body of evidence will be assessed (such as GRADE)	▬	▬	425 – 435

*** It is strongly recommended that this checklist be read in conjunction with the PRISMA-P Explanation and Elaboration (cite when available) for important clarification on the items. Amendments to a review protocol should be tracked and dated. The copyright for PRISMA-P (including checklist) is held by the PRISMA-P Group and is distributed under a Creative Commons Attribution Licence 4.0.**

From: Shamseer L, Moher D, Clarke M, Ghersi D, Liberati A, Petticrew M, Shekelle P, Stewart L, PRISMA-P Group. Preferred reporting items for systematic review and meta-analysis protocols (PRISMA-P) 2015: elaboration and explanation. BMJ. 2015 Jan 2;349(jan02 1):g7647.

For peer review only

Appendix C: Data Extraction Form

The below template will be used to extract data from included studies.

Inclusion/Exclusion form:

A. Reference details:

Reference details						
A1. Ref ID						
A2. 1st Author						
A3. Title of paper						
A4. Journal						
A5. Volume						
A6. Year of publication						
A7. Publication type	Paper1		Abstract2		Other3	
A8. Assessor's name	LK1		SR2		Other3	
A9. Date						

B. Study included in the review:

Yes1		No2	

Reason(s) for exclusion (if excluded):	Yes1	No2
B1. Non-human participants		
B2. Non-English language		
B3. Publication date		
B4. Study design and/or statistical analyses		
B5. Publication type		
B6. Selected on physical health problem		
B7. Selected on other developmental disorder		
B8. Selected on other mental health diagnosis		
B9. No risk factor measure		
B10. No disruptive behaviour outcome measure		
B11. Timing of measures		
B12. Duplicate (Insert Ref ID of other study)	Ref ID:	
B13. Other please specify:		

For peer review only

Data extraction form:

Reference details						
A1. Ref ID						
A2. 1st Author						
A3. Title of paper						
A4. Journal						
A5. Volume						
A6. Year of publication						
A7. Publication type	Paper$_1$		Abstract$_2$		Other$_3$	
A8. Assessor's name	LK$_1$		SR $_2$		Other $_3$	
A9. Date						

Study details												
B1. Name of study/cohort												
B2. Design	Cross-sectional$_1$		Prospective cohort$_2$		Retrospective cohort$_3$		Case-control$_4$		Linked admins$_5$		Other$_6$	
B2A. If other:												
B2B. Participants birth year, if given	Average$_1$		Max$_2$		Min$_3$		Other$_4$					
B3. Country	UK$_1$		USA$_2$		AUS$_3$		Other$_4$					
B3A. If other:												

Risk factor(s) measures included				
C1. Maternal smoking during pregnancy	Yes$_1$		No$_2$	
C2. Harsh, coercive discipline	Yes$_1$		No$_2$	
C3. Maltreatment	Yes$_1$		No$_2$	
C4. Divorce	Yes$_1$		No$_2$	
C5. Maternal adolescent motherhood	Yes$_1$		No$_2$	
C6. Parental depression	Yes$_1$		No$_2$	
C7. Parental antisocial behaviour	Yes$_1$		No$_2$	
C8. Parental drug use	Yes$_1$		No$_2$	
C9. Peer deviance	Yes$_1$		No$_2$	

For peer review only

C10. Poverty	Yes$_1$		No$_2$	
C11. Neighbourhood disadvantage	Yes$_1$		No$_2$	
C12. Hyperactivity / impulsivity	Yes$_1$		No$_2$	
C13. Low IQ / school achievement	Yes$_1$		No$_2$	
C14. Drug use	Yes$_1$		No$_2$	
C15. Resting heart rate	Yes$_1$		No$_2$	
C16. Skin conductance	Yes$_1$		No$_2$	
C17. Autonomic fear conditioning	Yes$_1$		No$_2$	
C18. Maternal alcohol use in pregnancy	Yes$_1$		No$_2$	
C19. Parental education	Yes$_1$		No$_2$	
C20. Family size	Yes$_1$		No$_2$	
C21. Bullying / peer victimisation	Yes$_1$		No$_2$	
C22. Stressful / adverse life events	Yes$_1$		No$_2$	
C23. Other:	Yes$_1$		No$_2$	

For each measure of risk factor(s)										
C24. Indicate which risk factor:										
C25. How was it measured:	Prospectively$_1$		Retrospectively$_2$							
C26. How was it ascertained	Parent$_1$		Self$_2$		Teacher$_3$		Observer$_4$		Others$_5$	
C27. Age(s) recorded (years)	Average$_1$		Max$_2$		Min$_3$		Other$_4$			
C28. Age recorded (groups)	Childhood$_1$		Adolescence$_2$		Adulthood$_3$					
C29. Above answers the same for all risk factors	Yes$_1$		No$_2$							

For peer review only

If no, copy and paste below as required										
C24. Indicate which risk factor:										
C25. Psychometric properties of measure given										
C26. How was it measured:	Prospectively[1]		Retrospectively[2]							
C27. How was it ascertained	Parent[1]		Self[2]		Teacher[3]		Observer[4]		Others[5]	
C28. Age(s) recorded (years)	Average[1]		Max[2]		Min[3]		Other[4]			
C29. Age recorded (groups)	Childhood[1]		Adolescence[2]		Adulthood[3]					

Disruptive behaviour measures included										
D1. Conduct problems	Child Behaviour Checklist (CBCL)[1] *or subset of CBCL e.g. Behavior Problems Index (BPI)		Eyberg Child Behavior Inventory[2]		Rutter Revised Preschool Scales[3]		DSM diagnostic items[4] *or measure that uses DSM criteria e.g. Child and Adolescent Psychiatric Assessment (CAPA) or Semi-Structured Assessment for the Genetics of Alcoholism (SSAGA)		Others[5]	
D1A. If other please describe										
D2. Conduct disorder	Diagnostic Interview Schedule for Children (DISC)[1]		Diagnostic Interview for Children and Adolescents - Revised (DICA-R)[2]		Schedule of Affective Disorders and Schizophrenia for School-Age Children—Present and Lifetime[3]		DSM diagnostic items[4] *or measure that uses DSM criteria e.g. Child and Adolescent Psychiatric Assessment (CAPA) or Semi-Structured Assessment for the Genetics of Alcoholism (SSAGA)		Others[5]	
D2A. If other please describe										
D3. Oppositional defiant disorder	The Oppositional Defiant Disorder Rating Scale[1]		Behavior Assessment System for Children: Second Edition (BASC-2)[2]		Child Behavior Checklist (CBCL)[3]		DSM diagnostic items[4]		Others[5]	

For peer review only

D3A. If other please describe										
D4. Antisocial personality disorder	Structured Clinical Interview for DSM-IV (SCID)$_1$		Psychopathy Checklist – Revised (PCL-R)$_2$		Mini International Neuropsychiatric Interview (MINI)$_3$		International Personality Disorder Examination (IPDE)$_4$		Others$_5$	
D4A. If other please describe										
D5. Other disruptive behaviour										
D6. Psychometric properties of measure given										
D7. How ascertained	Parent$_1$		Self$_2$		Teacher$_3$		Observer$_4$		Others$_5$	
D7B. If Other, please describe										
D8. Age(s) ascertained (years)										
D8B. Age of onset	Years$_1$		Months$_2$		Childhood$_3$		Adolescence$_4$		Others$_5$	
D9. Comordibities	Yes$_1$		No$_2$							
D10A. If Yes, please describe										
D11. Comments										

Available participant numbers						
E1. Baseline	Yes$_1$		No$_2$		If yes, number	
E2. Excluded	Yes$_1$		No$_2$		If yes, number	
E3. Refused	Yes$_1$		No$_2$		If yes, number	
E4. Lost to follow-up	Yes$_1$		No$_2$		If yes, number	
E5. Other losses	Yes$_1$		No$_2$		If yes, number	
E6. Included in analysis	Yes$_1$		No$_2$		If yes, number	
E7. All accounted for	Yes$_1$		No$_2$			
E8. Length of follow up						
E9. Procedures to deal with missing data						

For peer review only

Variable details						
F1. Risk factor	Continuous$_1$		Categorical$_2$		Other$_3$	
F1A. If other please describe						
F2. Disruptive behaviour	Continuous $_1$		Categorical$_2$		Other $_3$	
F3. Comments						

Details of study design and analyses						
G1. Quasi-experimental features	Adoption study$_1$		Fixed effect$_2$		Twin study$_3$	
	Propensity Score Matching$_4$		Sibling study$_5$		Regression discontinuity$_6$	
	Instrumental variable$_7$		Interrupted time series$_8$		Mendelian randomisation$_9$	
	Matching study$_{10}$		Experience sample$_{11}$		Ecological momentary assessment$_{12}$	
	Difference in difference$_{13}$		In vitro fertilisation$_{14}$		Polygenic risk score$_{15}$	
	Natural experiment$_{16}$		Matched control$_{17}$		Potential outcome$_{18}$	
	Balancing covariate$_{19}$		Controlled before and after$_{20}$		Inverse probability weight$_{21}$	
	Doubly robust regression$_{22}$		Selection model or selectivity model$_{23}$		Heckit model or Heckman sample selection$_{24}$	
	Selection correction$_{25}$		Two stage residual inclusion$_{26}$		Sharp design or fuzzy design$_{27}$	
	Forcing variable$_{28}$		Full information maximum liklihood$_{29}$		Natural control$_{30}$	
G1A. If other:						
G2. Included in analysis	Males and females$_1$		Males only$_2$		Females only$_3$	

G3. Model type	Linear$_1$		Logistic$_2$		Poisson$_3$		Survival$_4$		Other$_5$	

G4. List models presented incl. unadjusted				
G5. Confounders selected	Yes$_1$		No$_2$	
G5A. If yes, please describe				

Effect estimates									
Association estimated and which group e.g. M/F/All		Cross sectional; longitudinal; time-to-event	No. analysed	Effect estimate given? Y/N	If N, what are the qualitative findings?	If Y, what is the type of effect estimate, category comparison/value of unit change	Effect estimate	95% CI; SE; p	Confounders adjusted for
1									
2									
3									

For peer review only

Potential References Identified for Inclusion		
Reference	Included in database search	Not included in database search

For peer review only

For peer review only - http://bmjopen.bmj.com/site/about/guidelines.xhtml

Appendix D: [MODIFIED] Risk Of Bias In Non-randomized Studies – of **Exposures** (ROBINS-E) assessment tool

Specify a target experiment specific to the study ______________________________

Participant ______________________________

⎯ The protocol specified target experiment fully applies OR Experimental exposure ______________________________

Control exposure ______________________________

Specify the outcome

Specify which outcome is being assessed for risk of bias (typically from among those earmarked for the Summary of Findings table). Specify whether this is a proposed benefit or harm of exposure.

____

Is your aim for this study…?

- ⎯ to assess the effect of initiating intervention (as in an intention to treat analysis)
- ⎯ to assess the effect of initiating and adhering to intervention (as in a per-protocol analysis)
- ⎯ other (specify) ______________________________

Specify the numerical result being assessed

In case of multiple alternative analyses being presented, specify the numeric result (e.g. RR = 1.52 (95% CI 0.83 to 2.77) and/or a reference (e.g. to a table, figure or paragraph) that uniquely defines the result being assessed.

____

For peer review only - http://bmjopen.bmj.com/site/about/guidelines.xhtml

Preliminary consideration of confounders

Complete a row for each important confounding area (i) listed in the review protocol; and (ii) relevant to the setting of this particular study, or which the study authors identified as potentially important.

"Important" confounding areas are those for which, in the context of this study, adjustment is expected to lead to a clinically important change in the estimated effect of the exposure. "Validity" refers to whether the confounding variable or variables fully measure the area, while "reliability" refers to the precision of the measurement (more measurement error means less reliability).

For peer review only

**Confounding**				
**(i) Confounding areas listed in the review protocol**				
**Confounding area**	**Measured variable(s)**	**Is there evidence that controlling for this variable was unnecessary?**	**Is the confounding area measured validly and reliably by this variable (or these variables)?**	**OPTIONAL: Is adjusting for this variable (alone) expected to move the effect estimate up or down?**
			Yes/No/No information	Favour intervention / Favour control / No information
			Yes/No/No information	Favour intervention / Favour control / No information
**(ii) Additional confounding areas relevant to the setting of this particular study, or which the study authors identified as important**				

Confounding area	Measured variable(s)	Is there evidence that controlling for this variable was unnecessary?	Is the confounding area measured validly and reliably by this variable (or these variables)?	OPTIONAL: Is adjusting for this variable (alone) expected to move the effect estimate up or down?
			Yes/No/No information	Favour intervention / Favour control / No information
			Yes/No/No information	Favour intervention / Favour control / No information

* In the context of a particular study, variables can be demonstrated not to be confounders and so not included in the analysis: (a) if they are not predictive of the outcome; (b) if they are not predictive of exposure; or (c) because adjustment makes no or minimal difference to the estimated effect of the primary parameter. Note that "no statistically significant association" is not the same as "not predictive".

For peer review only

Preliminary consideration of criteria used to determine the accuracy of measurement of exposure and outcome

Complete a row for each measure listed in the study for the (i) exposure and (ii) outcome. Of the measures listed in the protocol, consider the sensitivity, specificity, and confidence in the methods used in the study.

**Accuracy of measurement**		
**(i) Exposure measurement method listed in the study**		
**Method of measurement**	**Measured exposure**	**Is the exposure measured validly and reliably by this method (or these methods)?**
		Yes/No/No information
**(ii) Outcome measurement method listed in the study**		
**Method of measurement**	**Measured exposure**	**Is the exposure measured validly and reliably by this method (or these methods)?**
		Yes/No/No information

For peer review only

For peer review only - http://bmjopen.bmj.com/site/about/guidelines.xhtml

Preliminary consideration of co-exposures

Complete a row for each important co-intervention (i) listed in the review protocol; and (ii) relevant to the setting of this particular study, or which the study authors identified as important.

"Important" co-interventions are those for which, in the context of this study, adjustment is expected to lead to a clinically important change in the estimated effect of the intervention.

**Co-exposures**		
**(i) Co-exposures listed in the review protocol**		
**Co-exposure**	**Is there evidence that controlling for this co-exposure was unnecessary (e.g., because it was not administered)?**	**Is presence of this co-exposure likely to favor outcomes in the experimental or the control group**
		Favor experimental / Favor comparator / No information
		Favor experimental / Favor comparator / No information
		Favor experimental / Favor comparator / No information
(ii) **Additional co-exposures relevant to the setting of this particular study, or which the study authors identified as important**		
**Co-exposure**	**Is there evidence that controlling for this co-exposure was unnecessary (e.g., because it was not administered)?**	**Is presence of this co-exposure likely to favor outcomes in the experimental or the control group**
		Favor experimental / Favor comparator / No information
		Favor experimental / Favor comparator / No information
		Favor experimental / Favor comparator / No information

For peer review only

Risk of bias assessment (cohort type studies)

Subsections	
1	Confounding
2	Selection of participants
3	Classification of exposure status
4	Departure from intended exposures
5	Missing Data
6	Measurement of outcomes
7	Selection of reported results
8	Overall judgement

Key	
NA	Not applicable
Y	Yes
PY	Potentially yes
PN	Potentially no
N	No
NI	No information

For peer review only

Confounding

**Bias due to confounding**		
**Question**	**Description**	**Response options**
1.1 Is there potential for confounding of the effect of exposure in this study?<br><br>If N or PN to 1.1: the study can be considered to be at low risk of bias due to confounding and no further signalling questions need be considered		Y / PY / PN / N
1.2 If Y or PY to 1.1: Was the analysis based on splitting follow up time according to exposure received?		NA / Y / PY / PN / N / NI

For peer review only - http://bmjopen.bmj.com/site/about/guidelines.xhtml

If N or PN to 1.2, answer questions 1.4 to 1.6, which relate to baseline confounding		
1.3. **If Y or PY to 1.2**: Were exposure discontinuations or switches likely to be related to factors that are prognostic for the outcome?		NA / Y / PY / PN / N / NI
**If N or PN to 1.3**, answer questions 1.4 to 1.6, which relate to baseline confounding		
1.4. Did the authors use an appropriate analysis method that adjusted for all the critically important confounding areas?		NA / Y / PY / PN / N / NI
1.5. **If Y or PY to 1.4**: Were confounding areas that were adjusted for measured validly and reliably by the variables available in this study?		NA / Y / PY / PN / N / NI
1.6. Did the authors avoid adjusting for post-exposure variables?		NA / Y / PY / PN / N / NI
**If Y or PY to 1.3**, answer questions 1.7 and 1.8, which relate to time-varying confounding		
1.7. Did the authors use an appropriate analysis method that adjusted for all the critically important confounding areas and for time-varying confounding?		NA / Y / PY / PN / N / NI
1.8. **If Y or PY to 1.7**: Were confounding areas that were adjusted for measured validly and reliably by the variables available in this study?		NA / Y / PY / PN / N / NI

**Judgement for bias due to confounding**		
**Risk of bias**		**Low / Moderate / Serious / Critical / NI**
Optional: What is the predicted direction of bias due to confounding?		Favours experimental / Favours comparator / Unpredictable

For peer review only - http://bmjopen.bmj.com/site/about/guidelines.xhtml

For peer review only

Selection of participants

Bias in selection of participants into the study		
**Question**	**Description**	**Response options**
2.1. Was selection of participants into the study (or into the analysis) based on participant characteristics observed after exposure status were measured?<br><br>**If N or PN to 2.1 go to 2.4**		Y / PY / PN / N / NI
2.2. **If Y/PY to 2.1:** Were the post-exposure variables that influenced selection associated with exposure?	Do not understand?	Y / PY / PN / N / NI
2.3. **If Y/PY to 2.2:** Were the post-exposure variables that influenced eligibility selection influenced by the outcome or a cause of the outcome?	Do not understand?	NA / Y / PY / PN / N / NI
2.4 Do start of follow-up and start of exposure coincide for most participants?		NA / Y / PY / PN / N / NI
2.5 If Y/PY to 2.2 and 2.3, or N/PN to 2.4: Were adjustment techniques used that are likely to correct for the presence of selection biases?	IVPW<br>MCS	NA / Y / PY / PN / N / NI

Judgement for selection of participants		
**Risk of bias**		**Low / Moderate / Serious / Critical / NI**
Optional: What is the predicted direction of bias due to selection of participants into the study?		Favors experimental / Favors comparator / Towards null /Away from null / Unpredictable

For peer review only - http://bmjopen.bmj.com/site/about/guidelines.xhtml

Classification of exposures

Bias in classification of exposures		
**Question**	**Description**	**Response options**
3.1 Is exposure status well defined?		Y / PY / PN / N / NI
3.2 Did entry into the study begin with start of the exposure?		Y / PY / PN / N / NI
3.3 Was information used to define exposure status recorded prior to outcome assessment?	Misclassification of exposure because you know the outcome [practical/actual bias]	Y / PY / PN / N / NI
3.4 Could classification of exposure status have been affected by knowledge of the outcome or risk of the outcome?	[potential bias]	Y / PY / PN / N / NI
3.5 Were exposure assessment methods robust (including methods used to input data)?		Y / PY / PN / N / NI

Judgement for classification of exposure		
**Risk of bias**		**Low / Moderate / Serious / Critical / NI**
Optional: What is the predicted direction of bias due to measurement of outcomes or exposures?		Favors experimental / Favors comparator / Towards null /Away from null / Unpredictable

For peer review only

For peer review only - http://bmjopen.bmj.com/site/about/guidelines.xhtml

Bias due to departures from intended exposures

Bias in departures from intended exposures		
**Question**	**Description**	**Response options**
4.1. Is there concern that changes in exposure status occurred among participants?<br><br>**If your aim for this study is to assess the effect of initiating and adhering to an exposure (as in a per-protocol analysis), answer questions 4.2 and 4.3, otherwise continue to 4.4 if Y or PY to 4.1.**		Y / PY / PN / N / NI
4.2. Did many participants switch to other exposures?		Y / PY / PN / N / NI
4.3. Were the critical co-exposures balanced across exposure groups?	If the exposure is time varying, did the authors target the effect of the initial exposure or the sustained exposure.	Y / PY / PN / N / NI
4.4. **If NY/PN PY to 4.1, or Y/PY to 4.2, or 4.3:** Were adjustment techniques used that are likely to correct for these issues?	Sustained treatment = focus; what methods were used?	NA / Y / PY / PN / N / NI

Judgement for classification of exposure		
**Risk of bias**		**Low / Moderate / Serious / Critical / NI**
Optional: What is the predicted direction of bias due to departures from the intended exposures?		Favors experimental / Favors comparator / Towards null

For peer review only

Missing data

**Bias due to missing data**		
**Question**	**Description**	**Response options**
5.1 Were there missing outcome data?		Y / PY / PN / N / NI
5.2 Were participants excluded due to missing data on exposure status?		Y / PY / PN / N / NI
5.3 Were participants excluded due to missing data on other variables needed for the analysis?		Y / PY / PN / N / NI
5.4 **If Y/PY to 5.1, 5.2 or 5.3:** Are the proportion of participants and reasons for missing data similar across exposures?		NA / Y / PY / PN / N / NI
5.5 **If Y/PY to 5.1, 5.2 or 5.3:** Were appropriate statistical methods used to account for missing data?		NA / Y / PY / PN / N / NI

**Judgement for missing data**		
**Risk of bias**		**Low / Moderate / Serious / Critical / NI**
Optional: What is the predicted direction of bias due to missing data?		Favors experimental / Favors comparator / Towards null /Away from null / Unpredictable

For peer review only

Measurement of outcomes

Bias in measurement of outcomes		
**Question**	**Description**	**Response options**
6.1 Could the outcome measure have been influenced by knowledge of the exposure received?		Y / PY / PN / N / NI
6.2 Was the outcome measure sensitive?	Sensitivity and specificity of the measurement<br>[a) were the psychometric properties of the outcome measure given]	Y / PY / PN / N / NI
6.3 Were outcome assessors unaware of the exposure received by study participants?		Y / PY / PN / N / NI
6.4 Were the methods of outcome assessment comparable across exposure groups?		Y / PY / PN / N / NI
6.5 Were any systematic errors in measurement of the outcome unrelated to exposure received?		Y / PY / PN / N / NI

Judgement for measurements of outcomes		
**Risk of bias**		**Low / Moderate / Serious / Critical / NI**
Optional: What is the predicted direction of bias due to measurement of outcomes?		Favors experimental / Favors comparator / Towards null /Away from null / Unpredictable

For peer review only - http://bmjopen.bmj.com/site/about/guidelines.xhtml

Selection of the reported results

Bias in selection of the reported result		
**Question**	**Description**	**Response options**
Is the reported effect estimate likely to be selected, on the basis of the results, from...		
7.1. ... multiple outcome *measurements* within the outcome domain?	If you have multiple outcome measurements, only picking those which are significant	Y / PY / PN / N / NI
7.2 ... multiple *analyses* of the exposure-outcome relationship?	If you have run multiple analyses, only picking those which are significant	Y / PY / PN / N / NI
7.3 ... different *subgroups*?	If you have many different subgroups (e.g. males vs females), only picking those which are signficant	Y / PY / PN / N / NI

Judgement for selection of the reported results		
**Risk of bias**		**Low / Moderate / Serious / Critical / NI**
Optional: What is the predicted direction of bias due to selection of the reported result?		Favors experimental / Favors comparator / Towards null /Away from null / Unpredictable

Overall judgement

Overall judgement		
**Risk of bias**		**Low / Moderate / Serious / Critical / NI**
Optional: What is the overall predicted direction of bias for this outcome?		Favors experimental / Favors comparator / Towards null /Away from null / Unpredictable

For peer review only - http://bmjopen.bmj.com/site/about/guidelines.xhtml
